# Complete Computational Exploration of Eight-Carbon Hydrocarbon Chemical Space

**DOI:** 10.1021/acs.joc.6c00623

**Published:** 2026-07-22

**Authors:** Stephen J. Harman, Kristaps Ermanis

**Affiliations:** School of Chemistry, 6123 University of Nottingham, University Park, Nottingham NG7 2RD, United Kingdom

## Abstract

Hydrocarbons are the most fundamental class of chemical species, but even the chemical space of those with 8 carbon atoms or less has not been explored exhaustively. Here we report a full enumeration and computational exploration of this space. Density functional theory based geometry optimization and energy calculations have identified all stable molecules within this space, forming a new database called CHX8. A universal strain value has been proposed and assigned to each of these molecules, acting as a proxy for synthesizability and providing a clear guideline of how synthetically plausible these molecules could be. This paper explores the limits of the chemical space within CHX8, with a focus on *trans*-fused, unsaturated, and anti-Bredt ring systems. We show that, contrary to prevailing wisdom, most of these unconventional structures should be synthetically accessible, with relative strain energies less than that of cubane. It is expected that this data set will inspire the synthesis of many new molecules with applications in various areas of chemistry, biology and materials science. The resulting data set also provides a valuable resource for the development of general and robust machine learning models.

## Graphical abstract

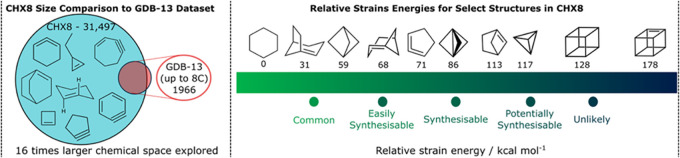

## Introduction

Exploring the breadth of chemical space has a long history. Since 1860 scientists and mathematicians have employed various approaches to calculate the magnitude and contents of chemical space.^1^ The first known attempt at this, made by Cayley using graph theory, showed that with 10 C atoms there were 150 different C_*x*_H_2*x*+2_ structural isomers. Later calculations examined the sheer size of chemical space,^2–5^ with one of the most renowned calculations, by Bohacek et al.,^6^ using combinatorial statistics to show that using only 30 total heavy atoms and 4 different elements more than 10^60^ molecules could be generated.

Recent efforts have shifted from estimating size to actually enumerating tractable regions, pioneering a “big data” approach to organic molecule space, most notably the generative databases (GDBs) for fragment and drug discovery.^7–23^ To mitigate against the combinatorial explosion, these enumerations were necessarily nonexhaustive and often applied relatively strict, general filters during their curation.

As a result while hydrocarbons are the most fundamental class of chemical species, even the chemical space of hydrocarbons with eight carbon atoms or fewer has not been explored exhaustively. In parallel, recent synthetic advances have repeatedly demonstrated that scaffolds once considered “unsynthesizable” may in fact be accessed and exploited (Figure 1a–c).^24–26^ While covering an impressive area of chemical space, libraries such as GDB-13 (fragments) and GDB-17 (drug-like molecules) excluded increasing numbers of structural features. Many of the excluded motifs correspond precisely to the classes highlighted in Figure 1—3D strained bioisosteres (a), cyclic allenes (b), and anti-Bredt frameworks (c).

**Figure 1:**
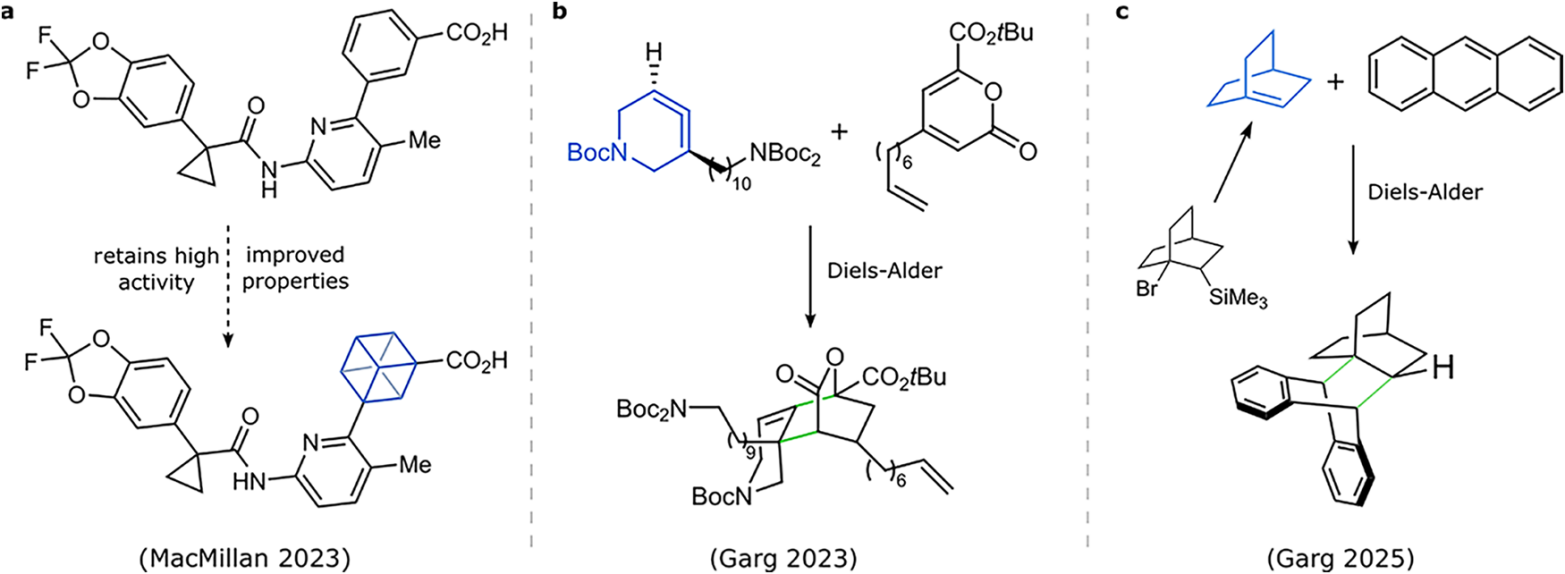
Contemporary drivers for exhaustive hydrocarbon maps. (a) Inclusion of 3D strained moieties in inhibitor molecules can improve pharmacokinetic properties^24^ (b) Cyclic allenes can be generated in situ to quickly build up complex hydrocarbon skeletons.^25^ (c) Anti-Bredt structures can be formed and used to build diverse frameworks using simple cyclization chemistry.^26^

As a particularly striking example, Garg and co-workers have demonstrated in situ generation and stereospecific trapping of cyclic allenes (Figure 1b) to enable the total synthesis of lissodendoric acid A and develop catalytic annulations that rapidly build complexity from these fleeting intermediates.^25,27^ They have also provided a general solution to access and intercept anti-Bredt olefins, long deemed inaccessible under Bredt’s rule (Figure 1c).^26^ Furthermore, they expanded the toolbox with nitrogen-containing strained cyclic 1,2,3-trienes.^28,29^ Together these advances motivate exhaustive, bias-minimized maps of hydrocarbon space that deliberately include strained and unconventional motifs, both to inspite new scaffold applicated (e.g., as bioisosteres)^24,30–33^ and to provide more representative training data for machine learning models, improving reliability outside of “comfortable” regions of chemical space.

Here, a full enumeration, mapping and density functional theory (DFT) optimization of hydrocarbon chemical space, including diastereoisomers, is reported for up to 8 carbon atoms. With no known omissions or deliberate rules for the exclusion of structural features, this represents the most complete collection of closed-shell hydrocarbon structures to date. We further introduce a universal relative strain energy (RSE) metric, referenced to cyclohexane, and assign a value to every molecule as a proxy for synthesizability, providing clear guidelines for synthetic plausibility. We particularly focus on strained unsaturated cyclic hydrocarbons as these have recently seen high-profile studies from the synthetic community, and, at the same time, have been largely omitted in previous chemoinformatic and computational enumerations.

## Methods

### General

Throughout this investigation calculations were performed using DFT with the PBE0 functional, D4 dispersion correction and def2-TZVP basis sets in the gas phase.^34–37^ This level of theory and basis is known to be accurate for geometry and frequency calculations at a reasonable computational cost.^38^ The calculations were run using Orca 5.0.4 software.^39,40^ Structure visualization was performed using Jmol 14.32, Avogadro 1.99 and Maestro 2023–3.^41–43^

### Enumeration and Structure Generation

Graph generation was performed using the GENG program from the NAUTY package, to give the graphs for all structures for C1 to C8.^44^ Custom Python scripts were used to convert the graphs to saturated structures before diastereoisomers, and unsaturated molecules were also generated (Figure 2). The 2D structures are stored using International Chemical Identifier (InChI) and Simplified Molecular Input Line Entry System (SMILES) formats. 3D structures were generated using RDKit 2022.9.5 and OpenBabel 3.1.1 Python packages.^45–48^ This process is discussed in more detail in Section 1 of the Supporting Information.

**Figure 2:**
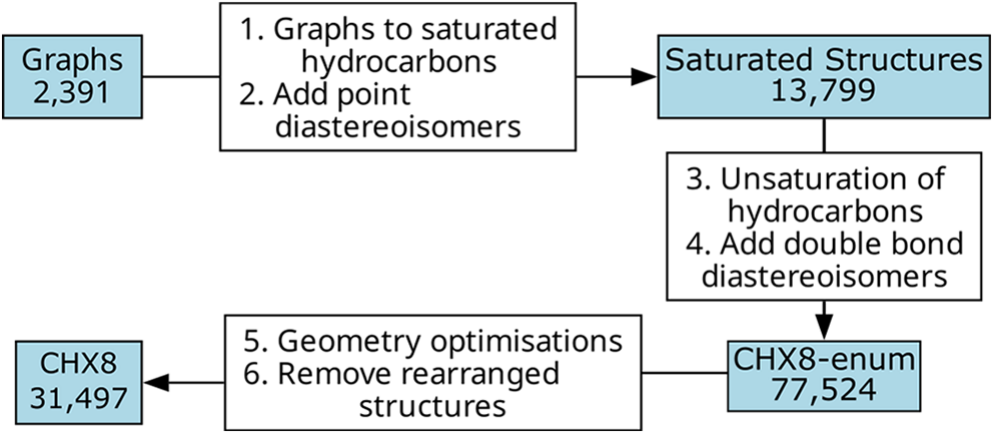
Workflow followed in the generation and optimization of the CHX8 database.

### Relative Strain Energy

Strain is a fundamental and important concept in organic and synthetic chemistry, allowing for the estimation of molecular stability and reactivity.^49–52^ It is the excess energy resulting from structural distortions and steric interactions relative to a reference structure considered to have zero strain.^53^ Here a generalized expansion of the methodology established by Dudev and Lim^54^ is employed, allowing for the calculation of the relative strain energy (RSE) of any hydrocarbon relative to a zero-strain reference, here cyclohexane. Equation 1 summarizes this approach.

1 $$RSE=E_{n}-E_{r}-\left(c_{n}-c_{r}\right)E_{CH_{2}}+E_{unsat}$$

In this work RSE is defined as the energetic difference between the system of interest (*E_n_*) and the reference (*E_r_*). This equation effectively constructs a virtual isodesmic reaction for each molecule. By explicitly accounting for the difference in the carbon count, (*c*_*n*_–*c*_*r*_), and degree of unsaturation, *E*_unsat_, we ensure that RSE only represents the geometric strain of the system, independent of stoichiometry or functional group density. This allows for destabilizing interactions, such as steric congestion or bond twisting, to be identified alongside electronically stabilizing interactions, such as hyperconjugation and aromaticity. Details on the values of these correction terms and method benchmarking are provided in SI 4.1–4.2.

## Results and Discussion

### Generation and Optimization of CHX

The CHX8-enum data set contains all mathematically feasible hydrocarbons with up to eight carbon atoms, detailing the 77,524 unique structures. During the DFT geometry optimizations some of these molecules underwent structural rearrangements, resulting in a changing σ-framework between input and output structures. CHX8-enum structures that were stable to optimization formed the final CHX8. This data set represents 41% of the preliminary CHX8-enum data set and contains the 31,447 structures which were stable to optimization (Table 1). Analysis of the rearranging structures was also performed to ensure that all the resulting structures had already been generated, and no new compounds were identified. (SI 2.1–2.2) The data set has been uploaded to the Nottingham Research Data Management Repository (10.17639/nott.7626).

**Table 1:** Saturated and Unsaturated Isomers Generated within CHX-enum and the Resulting Stable DFT-Optimized Geometries for Each Hydrocarbon (CHX8), Separated by Heavy Atom Count (HAC)

HAC	graphs	saturated	unsaturated	CHX-enum	CHX8
1	1	1	0	1	1
2	1	1	2	3	3
3	2	2	7	9	8
4	6	7	31	38	30
5	21	25	138	163	117
6	78	114	753	867	522
7	353	746	4939	5685	2917
8	1929	12,903	57,856	70,758	27,899
total	2391	13,799	63,726	77,524	31,497

### CHX vs Other Data Sets

As an initial check on the quality of this data set, a comparison of the data set with other enumerations was performed, particularly against the most notable previous enumeration of chemical space: the GDB data sets generated by Reymond et al.^8,9,22,23,55–60^ GDB-17 represents the most expansive enumeration to date, describing over 166 billion small-to-moderately sized organic molecules, however considering only 8 carbon hydrocarbons, GDB-13 is a larger collection with 1966 structures. All of GDB-13 hydrocarbons were found within the CHX-enum data set, however some of these were not stable to optimization and thus are not in the final CHX8. CHX8 represents a space more than 16 times larger than GDB-13 (1966) (Figure 3).

**Figure 3:**
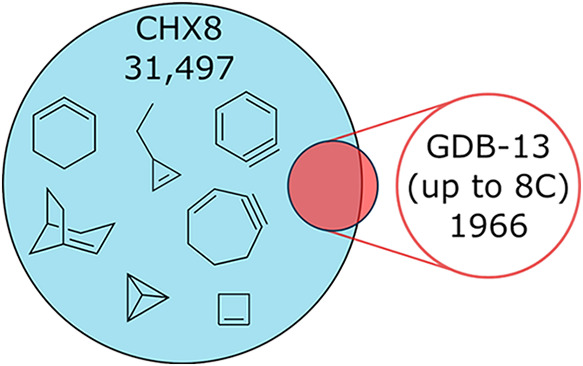
Venn-diagram representation of the relative sizes of GBD-13 and CHX8 with selected molecules in CHX8 but not in GDB-13. Some of GDB-13 molecules were found to be unstable to geometry optimization in this study, so they do not appear in CHX8.

Comparison of CHX8 to public databases of chemical space was also performed. The aim of this was to examine the scope of chemical space that has been explored up to this point. It was found that less than 44% of CHX8 was known in PubChem, and analysis of the REAXYS database showed only 25% of CHX7, that is the CHX8 data set with up to seven C atoms, was commercially available. (SI 5.2–5.4). The larger eight-carbon chemical space cannot be easily checked in a representative way, however it is expected that even less of this space would be commercially available. Comparison to existing databases and other enumerations demonstrates that CHX8 represents an area of chemical space that has previously been incompletely explored both computationally and synthetically.

### Strain in CHX8

Within the chemical space uniquely documented in CHX8 there are many systems that exhibit polycyclic ring systems and complex molecular architectures, with correspondingly large relative strain energy. While in previous enumerations highly strained structures have been excluded from consideration,^8,9,23^ here they are included and examined in quantitative detail. This is achieved through assignment of an RSE value for all structures in the DFT-optimized CHX8 data set. Details of the development and optimization of the method are described in detail in Section 4 of the Supporting Information.

To examine how strain varies across CHX8, we mapped both previously synthetically accessed and hypothetical structures onto the RSE scale (Figure 4).^61–63^ These highlighted molecules are used as reference points for the RSE values calculated in this study to serve as a proxy for synthesizability. Structure **5** (C6, Figure 4) was identified as the highest RSE structure with synthetic precedent.^63^ This structure has been synthesized on a metal surface and was detected using atomic force microscopy. To the best of our knowledge, **5** has the highest RSE of any previously synthesized molecule in CHX8 (201.4 kcal mol^–1^) and so it is suggested that all structures with a lower RSE than **5** might be synthetically achievable in some capacity. Using this definition of synthetic accessibility, over 90% of the structures which are novel in our data set should be considered in principle synthesizable and therefore experimentally relevant. Analysis of the application of the strain scale to CHX8 demonstrated in Figure 4 was then shifted toward more traditional structures, both with and without synthetic precedent.

**Figure 4:**
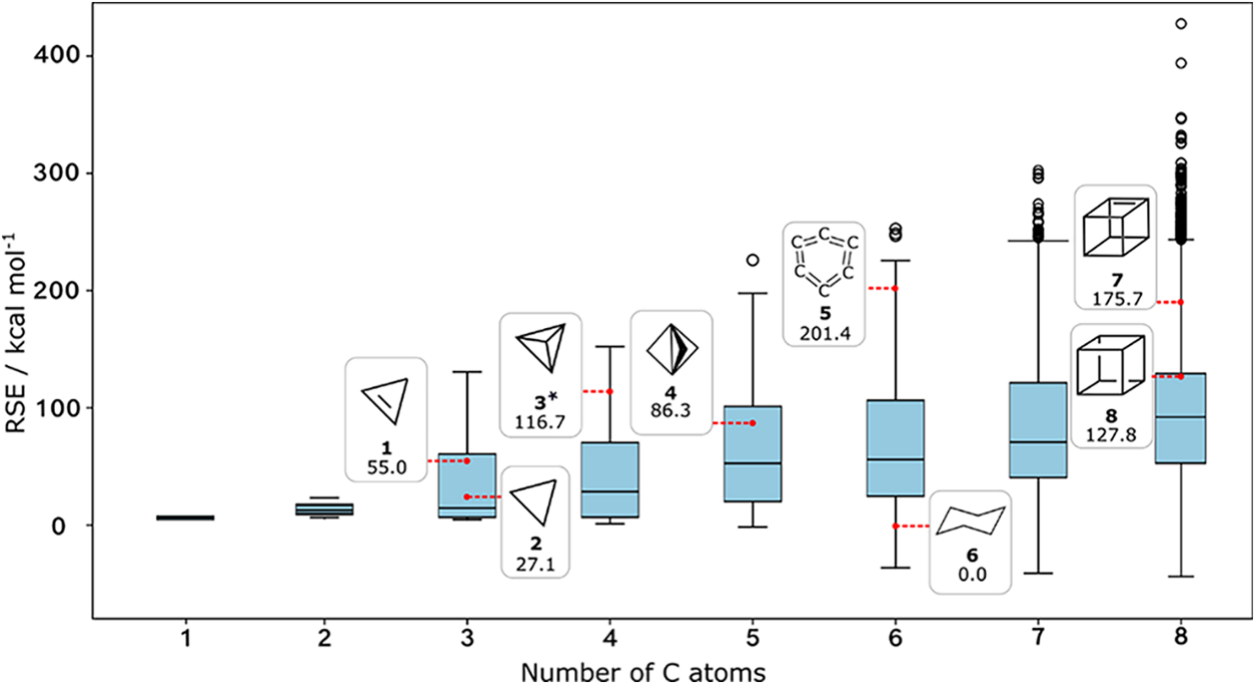
Distribution of relative strain energies for CHX8 structures as a function of the number of C atoms. Box-and-whiskers plot illustrates the distribution of molecules on the RSE scale. Reference structures, alongside structures of interest are highlighted. Asterisked molecules are as-yet unsynthesized and all others have been observed or inferred synthetically.^61–63^

Cubane (**8**) and tetrahedrane (**3**) are structurally similar compounds, each being the molecular equivalent of a platonic solid, and both exhibiting potentially exciting properties.^24,30,31^ Substituted tetrahedranes have been previously synthesized, demonstrating higher thermal stability than would be expected of these compounds.^64,65^ Previous computational studies on the strain energies of **3** and **8** agree with the RSE calculations performed here, finding that **3** has a lower RSE than **8**, suggesting that the synthesis of unsubstituted tetrahedrane should, in principle, be possible.^66,67^

### Alkenes in Rings

CHX8 and the universal strain scale allow the probing of other aspects of stability and synthetic feasibility in molecular features. While *cis-*double bonds are ubiquitous in all ring systems, *trans-*double bonds in rings are a less explored motif. While it is widely assumed that the smallest ring that may contain a *trans-*double bond is cyclooctene,^68^ there is some experimental evidence that smaller cyclic *trans*-alkenes might be feasible.^69–73^ Here the question of the smallest accessible *trans*-alkene is re-examined computationally.

Study of the *trans-*double bond containing ring structures within CHX8 showed that any six membered ring or larger was able to support a single double bond in the *trans-*configuration. This was determined by achieving a stable structure without a change in σ-framework or stereochemistry during gas phase DFT geometry optimization (Figure 5). This aligns with experimental investigations of these systems that have accessed *trans-* **11** and *trans-* **12** either as intermediates in synthesis or as bench stable complexes.

**Figure 5:**
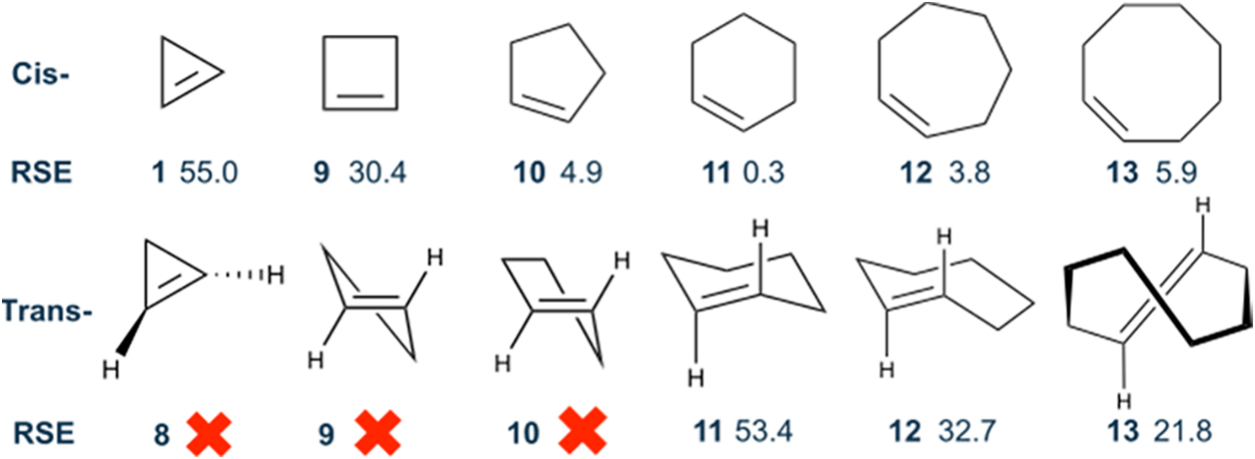
*cis*- and *trans*-ring double bonds generated in CHX-enum, alongside their RSE in kcal mol^–1^ where they were stable to optimization.

Focus was then directed toward the capacity of these systems to host more than one trans-double bond. It was observed that the relative position of the introduced double bonds was fundamental to the stability of the generated structures, and an exemplary case is presented here. *trans*-Cyclohexadiene has two nonallene positional isomers (Figure 6). As both structures are conformationally locked in a chairlike structure by the double bonds, it was expected that these very similar geometries would behave in the same way. However, only one structure was found to be stable to optimization.

**Figure 6:**
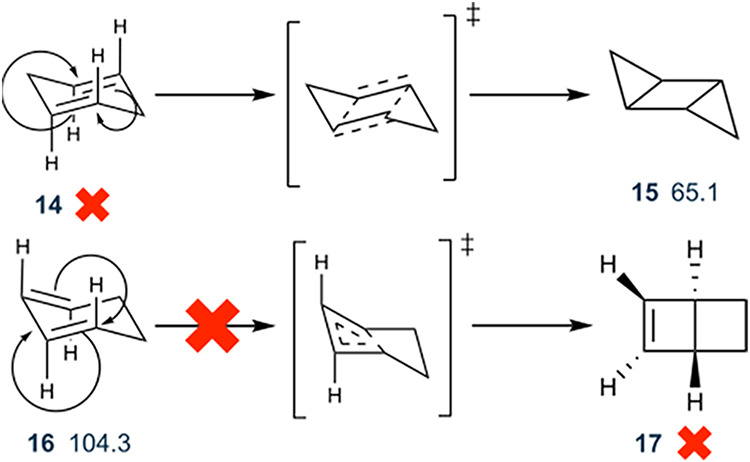
Nonallene configurational isomers of E,E-cyclohexadiene. The mechanism for rearrangement in the 1,4- and 1,3-isomers alongside the RSE of the product in kcal mol^–1^ where relevant.

In **14** the two trans-double bonds are parallel in the ring. This results in a structure optimally oriented for a thermally accessible 4π-electrocyclization to occur, forming **15**. In **16**, the geometric relationship between the two double bonds would result in the formation of trans–trans-bicyclo[2.2.0]­hex-2-ene, **17**. This contains the trans-bicyclo[2.2.0]­hexene moiety, found here to be unstable, and so it is expected that the energy barrier for cyclization of **16** would be very large. This highlights the dependence of stability on both internal strain, and the presence of accessible low-energy pathways for its release.

### Alkynes and Allenes in Rings

Alkynes and allenes are prevalent structures in CHX8, with 37% of the data set being cyclic alkynes or cyclic allenes. These compounds are commonly employed in strain-release syntheses in a range of organic processes, and when included in ring systems offer the same benefits to synthetic reactivity as in acyclic systems, for example, being useful for building complex structures quickly.^28,74–77^ These motifs were largely excluded in the GDB-17 database. Here, the range of moderate to highly strained cyclic structures contained within the data set is presented in Figure 7.

**Figure 7:**
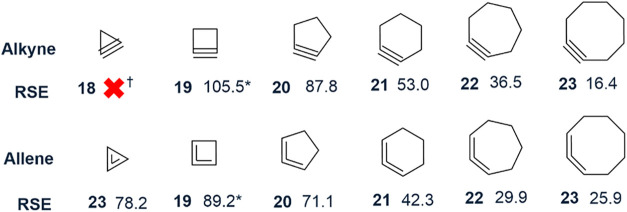
Cyclic alkyne and allene structures identified in CHX8 alongside their RSE in kcal mol^–1^ where they are stable to optimization. † See SI 6.1 *RSE values given for the triplet multiplicity structures, which were stable to optimization, where singlet structures were not.

Many studies looking at the reactivity of cycloalkynes are limited to medium sized rings, with smaller rings often going unmentioned or not being explored fully.^78–80^ A study by Goetz et al. predicted the accessibility of different heteroaryne structures proposed that some aryne structures were ‘inaccessible’ because their strain energies were too high.^81^ Recently this was shown not to be the case, and these ‘impossible arynes’ were accessed through coordination to transition metal atoms.^82^ Our computational results in Figure 7 align with this and show that all cycloalkynes, except cyclopropyne and all cyclic allenes are feasible with strain energies less than that of cubane. This development showcases the importance of thorough explorations of chemical space without strong preconceptions of what may be plausible or useful.

### Bredt and Anti-Bredt Structures

Bredt’s rule is a convention in organic chemistry that states that introducing an unsaturation to the bridgehead atom of a bridged molecule is either impossible or very difficult due to the strain that is generated.^83^ One approach for the classification of synthetic accessibility of anti-Bredt structures was by Fawcett who introduced an “*S*” parameter. This is equal to the number of nonbridgehead atoms in the ring system. Initially, Fawcett suggested that anti-Bredt structures with *S* values of 9 or higher should be synthetically accessible.^84^ Subsequently, lower values for this parameter S (7 and even 4) have been accessed as intermediates in synthetic pathways.^85,86^

Saturated bridgehead structures are hereafter referred to as bridged structures, while structures with an unsaturated bridgehead atom, breaking Bredt’s rule, are called anti-Bredt structures. Due to the disfavored alkene geometry at this bridgehead position anti-Bredt structures are generally highly strained, and this is often related to the inaccessibility of *trans-*double bond formation in the ring systems. In Alkenes in Rings Section it was demonstrated that this functionality is more accessible than is commonly believed. Furthermore recent studies of anti-Bredt systems have accessed structures that are predicted to be inaccessible by Bredt’s rule.^84,86^

Within CHX8 there are seven hydrocarbon skeletons that include one bridging section of any size (Figure 8). Of these hydrocarbon frameworks, 13 of the 14 combinatorially possible unsaturated anti-Bredt skeletons are found to be stable within CHX8. Four of these have been seen synthetically, often through trapping experiments.^86–88^

**Figure 8:**
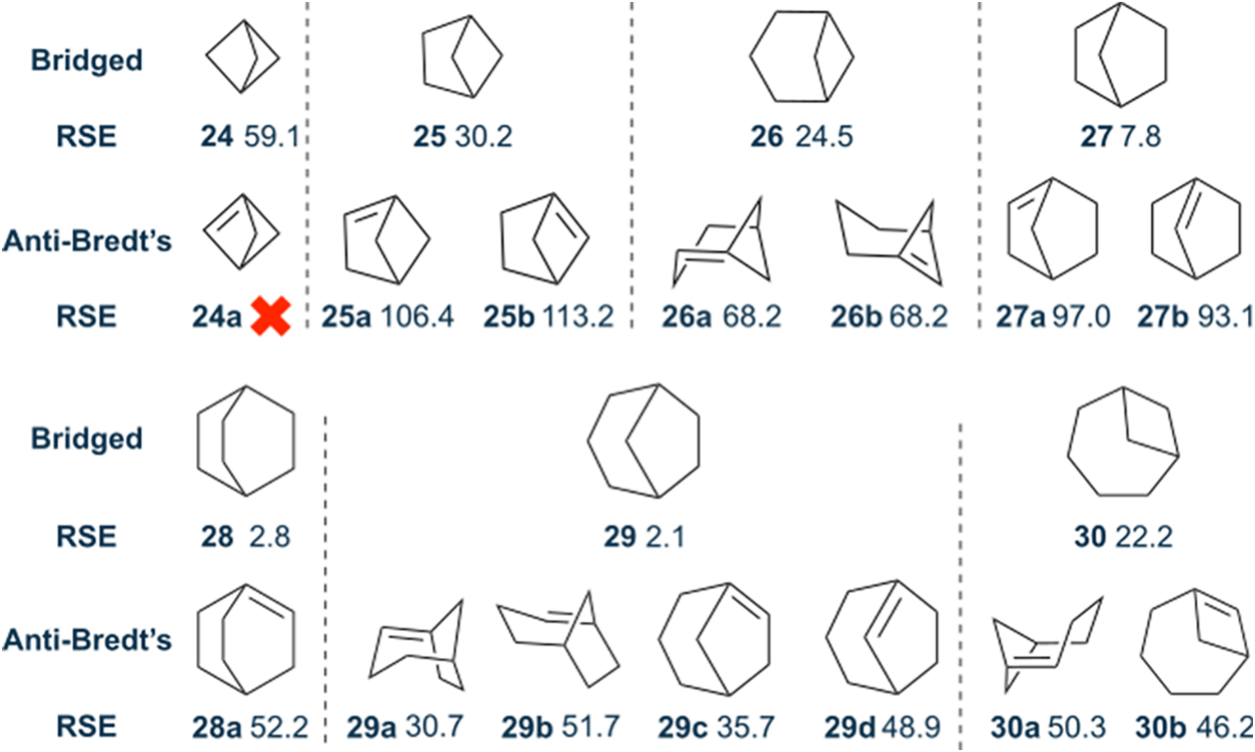
Bridgehead containing hydrocarbon skeletons in CHX8, with all anti-Bredt derivatives, alongside their RSE in kcal mol-1 where they are stable to optimization.

The smallest hydrocarbon possessing the bridgehead alkene is **24**. When this small bridgehead molecule is unsaturated to generate **24a**, the resultant strain energy causes it to undergo a rearrangement during optimization (Figure 9). This occurs via a heterolytic ring opening generating a zwitterion, which quickly rearranges to give the carbene structure **31** which has experimental precedent.^89^

**Figure 9:**
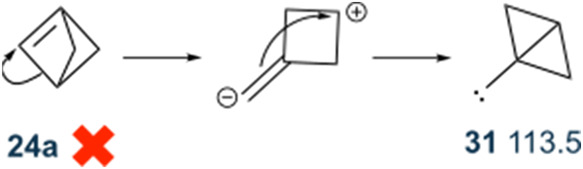
Rearrangement of 24a during optimization to give 1-methylene-bicyclo[1.1.0]­butane, alongside the RSE of this structure in kcal mol^–1^.

The findings in CHX8 suggest that, in agreement with experimental results, structures with S values of 4 or higher are stable to optimization. Interestingly the S value does not act as a strong predictor of RSE in bridged structures. (SI 6.2.1)

By introducing various substituents to the structures presented in Figure 8, CHX8 contains over 200 anti-Bredt structures, demonstrating the diversity of frameworks, alongside how their modification may be leveraged to manipulate their RSEs (SI 6.2.2). This research and recent experimental work highlights that while general synthetic accessibility rules are useful, they are at best guidelines, and exciting structures and reactivity can be found among the exceptions.

### Trans-Fused Rings in Polycyclic Hydrocarbons

Small rings are ubiquitous throughout this chemical space as noted in previous studies.^23^ However, in these previous investigations polycyclic molecules with small rings were excluded by design. While *cis-*ring junctions are known for all bicyclic systems, there are only few studies examining the lower ring size limit for even simple, unsubstituted *trans-*ring junctions, and it is generally accepted that the smallest *trans*-ring junction is the 6,4-bicyclic ***trans-*** **39**.^68^ Within CHX8 all possible simple fused ring systems with up to eight carbon atoms are present and have been investigated (Figure 10).

**Figure 10:**
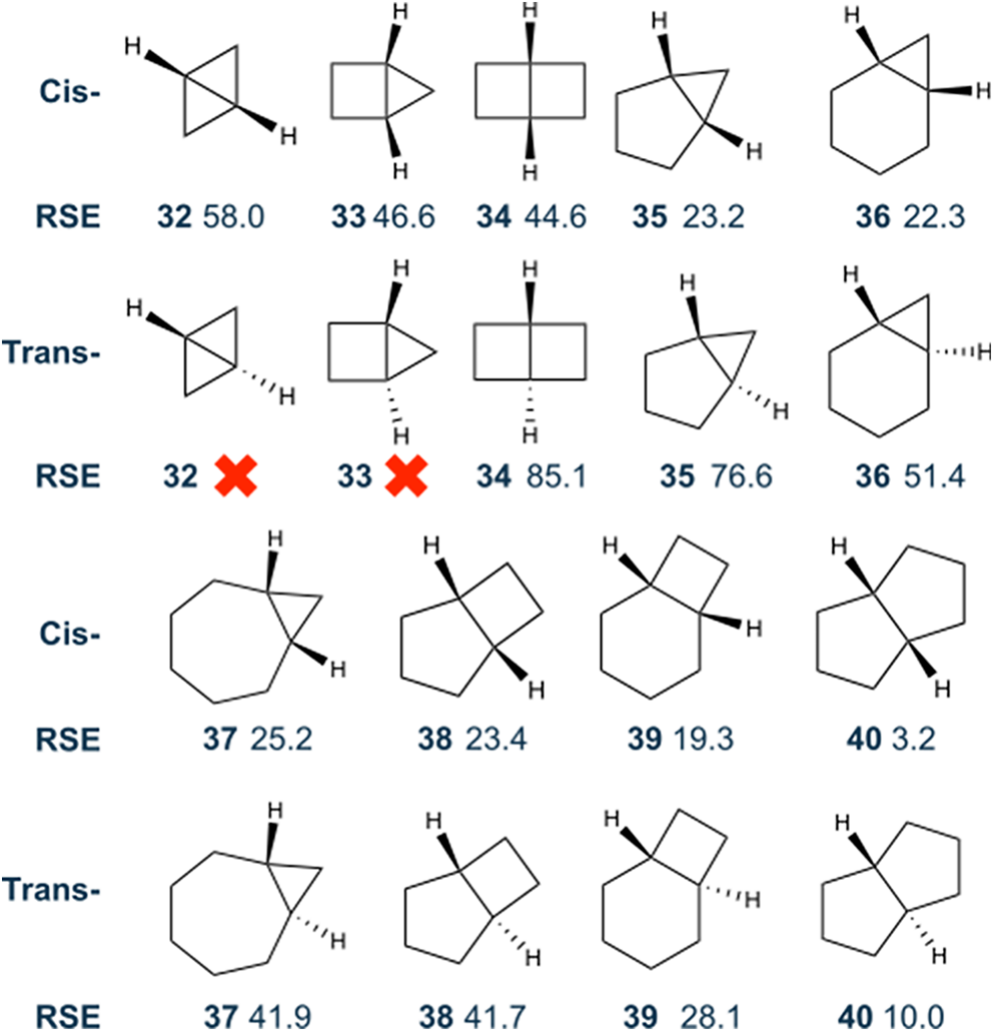
*cis-* and *trans-*ring junctions generated in CHX-enum, alongside their RSE in kcal mol^–1^ where they were stable to optimization.

The smallest theoretically possible *trans-*ring junctions, ***trans*** **-32** and ***trans-*** **33** were found to spontaneously rearrange during optimization, due to their highly strain geometries. ***Trans-*** **32** rearranged through a heterolytic bond fission mechanism to produce a zwitterionic cyclobutane derivative. ***Trans-*** **33** rearranges initially in the same manner, with a heterolytic ring opening followed by a 1,2-hydride migration, resulting in the formation of **10** (Figure 11). This is a known thermally accessible reactivity that has been exploited in ***cis-*** **33**.^90^

**Figure 11:**
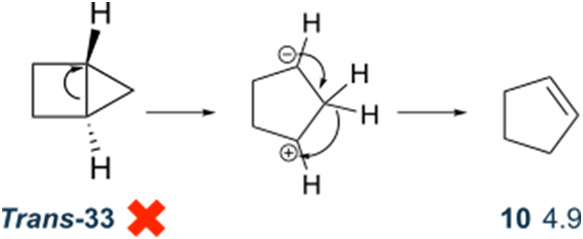
Rearrangement of *trans-*33 to give 10, alongside the RSE of 10 in kcal mol^–1^.

All larger fused ring structures are based on structures with six or more total ring atoms.^91^ These all successfully optimized to structures with a trans-geometry, and the resulting strain values, while generally high, are in the potentially synthesizable region (<130 kcal mol^–1^). Thus it is proposed that a fundamental lower limit for molecules with trans-ring junctions is either; (i) a 3-membered ring trans fused to a ring of five or more atoms, or (ii) a 4-membered ring trans-fused to another 4-membered ring.

To understand how inclusion of multiple ring junctions on the same central rings affects stability, additional investigation was undertaken. From this, rules for inclusion of trans-fused rings are devised, details of which are presented in SI 6.3–6.4.

## Conclusion

CHX8 is the first data set fully accounting for both; (i) strained unusual molecular architectures, that have been excluded early on in other investigations, and (ii) diastereoisomerism, a feature that has not been exhaustively explored previously.

This exploration of hydrocarbon chemical space uncovers far broader structural diversity than prior efforts—CHX8 exceeds the corresponding GDB-13 region by more than a factor of 16,^9^ delivering markedly wider coverage. This expanded data set offers a more comprehensive map of hydrocarbon space, enabling the training of stronger, more reliable machine-learning models.

CHX8 is a future-proofed database as no structures stable in DFT optimization have been filtered out. This results in a data set containing structures that are accessible by current standards but also reports novel structures, which may become accessible in the future as synthetic paradigms evolve and develop.

The application of a generalized hydrocarbon strain equation has also been demonstrated and shown to generate reliable results that agree with experimental and computational values in the literature.

Additionally, by using relative strain energy as a proxy for synthesizability, the potential limits of hydrocarbon synthetic accessibility have been re-examined. Specifically, we show computationally that

1. All *trans*-cycloalkenes in 6-membered rings or larger should be synthetically feasible;
2. All cyclic alkynes and allenes should be synthesizable either in their singlet or triplet states;
3. All but [3,3]- and [3,4]- trans-fused unsubstituted rings should be accessible.
4. All but one of theoretically possible anti-Bredt structures should be feasible, with relative strain energies for all being less than that of cubane.

This work underscores the importance of small molecule architectures in the scope of larger-scale chemical space exploration, while also revealing new regions which as yet remain unexplored synthetically. It is anticipated that this data set and the guidelines derived from it will find applications in organic synthesis, medicinal and biological chemistry, and other areas. Efforts to expand this approach to other areas of chemical space are underway in our laboratory.

## Supplementary Material



## Data Availability

The data underlying the study are available in the published article, in its Supporting Information, and openly available in the Nottingham Research Data Management Repository, at 10.17639/nott.7626.

## Notes

Additional discussion referenced in the text, generation optimization and the presentation of the dataset, the benchmarking and discussion of our RSE methodology, additional comparison between existing datasets and CHX8, visualization of CHX8 using dimensionality reduction; the CHX8 dataset: SMILES and InChIs for optimized geometries, all mol input and xyz, geometry optimization and frequency output files; the SMILES and InChI strings for the molecules generated prior to stereoisomer generation (PDF)
