# Supplementary material for: Complete Computational Exploration of Eight-Carbon Hydrocarbon Chemical Space
Source: J Org Chem. 2026 Jul 22;91(30):10365–73. doi: 10.1021/acs.joc.6c00623 (PMC13435312; doi:10.1021/acs.joc.6c00623)
Supplement: Supplementary file 1 [file jo6c00623_si_001.pdf]

Supporting Information

Complete Computational Exploration of Eight-Carbon Hydrocarbon Chemical Space

Stephen J. Harman, Kristaps Ermanis*
AUTHOR ADDRESS: School of Chemistry, University of Nottingham, University Park, NG7 2RD, United Kingdom

1. Generation of Dataset S2
   1.1 Generation of Saturated Structures S2
   1.2 Generation of Unsaturated Structures S3
   1.3 Filtering, Elimination and Lost Structures S4
2. Optimisation and Separation of Dataset S7
   2.1 General Details of Optimisation S7
   2.2 Separation Details S7
3. Navigating the Dataset S9
   3.1 File contents and hierarchy S9
   3.2 Identifying geometries before and after optimisation S9
   3.3 Structure searching within the dataset S9
4. Validation of the Approach for RSE Calculation S11
   4.1 The RSE equation S11
   4.2 A series of benchmarking values S12
   4.3 The Effect of Me-Substitution on the RSE of Small Rings S13
5. Comparison to Databases/Datasets S15
   5.2 Comparison to VQM24 S15
   5.3 Comparison to PubChem S15
   5.4 Comparison to REAXYS S15
6. Further Discussion from the Main Text S16

6.1 On the stability of cyclic-allene and alkyne hydrocarbons S16
6.1.1 Small Ring Allenes and Alkynes
6.1.2 On the stability of cyclo-propyne
6.2 On the stability of anti-Bredt structures S17
6.2.1 Correlation between S value and RSE
6.2.2 Improving the stability of anti-Bredt structures.
6.3 Trans-ring Junctions S19
6.3.1 Multiple ring junctions on the same central ring
6.3.2 Trans-ring junctions – Rules for Synthesis and Stability
7. Visualisation of CHX8 S21

1. Generation of the Dataset

Graph generation was performed using the GENG program from the NAUTY package, to give the graphs for all structures for C1 to C8.[1] Custom Python scripts were used to convert the graphs to saturated structures before diastereoisomers, and unsaturated molecules were also generated. The 2D structures are stored using International Chemical Identifier (InChI) and Simplified Molecular Input Line Entry System (SMILES) formats. 3D structures were generated using RDKit 2022.9.5 and OpenBabel 3.1.1 Python packages.[2–5] (see Figure S1 and SI 1.1-1.3 for further details.)

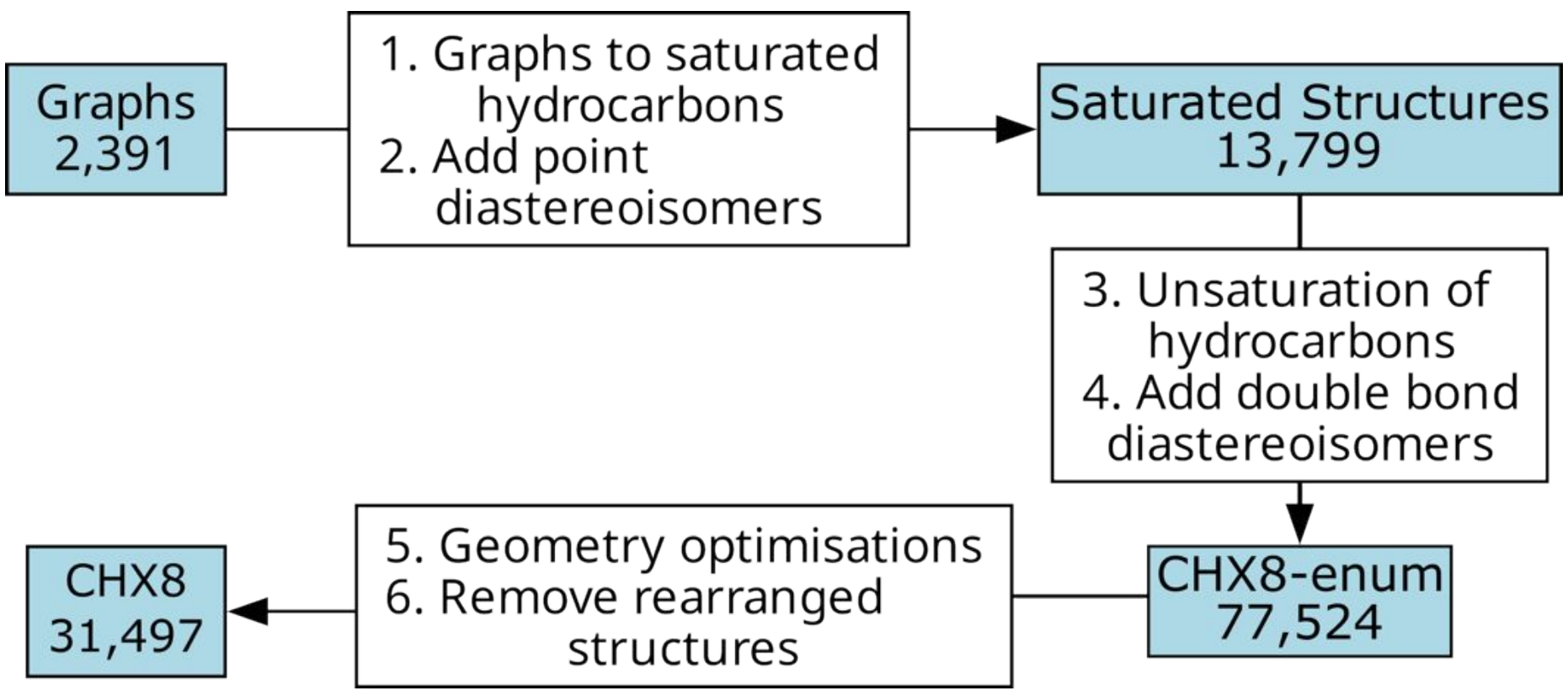

***Figure S1**: Workflow followed in the generation and*

1.1 Generation of Saturated Hydrocarbons

The core graph skeletons that provided all saturated structures contained within CHX8-enum were generated using the GENG software from NAUTY. This generated all 1-8 nodal graphs that had with between 1-4 edges per node. No filtering processes were used to remove any graphs from the dataset, and checks were performed to ensure that no disconnected graphs were generated, that is a graph that is made up of any number of smaller, disconnected graphs. This resulting in the graph structures, which formed the basis of this enumeration.

Table S1: Number of graphs with each number of nodes generated.

Nodes	Number of Graphs
1	1
2	1
3	2
4	6
5	21
6	78
7	353
8	1929

These graphs were then converted to RDKit molecule objects, SMILES, and InChI strings. The InChI string for each of the structures was then checked. Largely this was done to prevent generation of duplicate structures. If the InChI of all previously generated structures were known, comparison to any newly generated InChI prevented inclusion of the same structure twice. Another benefit of the InChI strings is the inclusion of the chiral information layer. This layer is always present in structures that can exhibit point diastereoisomerism and is identified as the "/t" section of the InChI string. After the chiral layer there is also an "/m?" and "/s?" label that may be included, these give information regarding which enantiomer of the structure is presented, where this is relevant (Table S1).

Table S2: The InChI strings for 1,2-dimethylcyclopropane with the different chiral layers describing the relative orientation of the methyl substituents.

InChI=1S/C5H10/c1-4-3-5(4)2/h4-5H,3H2,1-2H3/t4?,5?/m0/s1	
Chiral Tag	2D Geometry
/t4-,5-	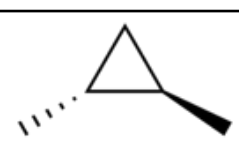
/4-,5+	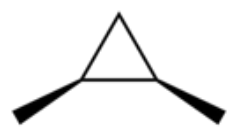

Approaches for generation of diastereoisomers available in RDKit were initially considered. However, these proved to be unreliable, and failed with less conventional geometries and having internal, hard-coded limits that prevents the generation of many small trans-ring junctions which are required for the enumeration. Consequently, an alternative method was developed.

This approach used the chiral, "\t" layer in the InChI strings of generated structures. On the initial pass, structures with the chiral tag were separated from those that did not. These structures were then passed through an iterative process, by which all possible InChI descriptors were produced. These were then canonicalised and duplicates were excluded. Some duplicates were not successfully detected and therefore persist in the dataset. This occurs as the enantiomer layer in the InChI string is not always kept the same during canonicalisation, and so duplicate structures cannot be identified trivially.

The complete list of canonicalised, saturated hydrocarbon InChIs, now including hydrocarbons was then converted to 3D structures through OpenBabel's "--Gen3D" function and the MMFF94 force field. This method was chosen as it gave the fasted structure generation results and resulting in the generation of the fewest zero coordinate structures (S.I: 1.3) across the methods tested and available. These factors were chosen for the benchmarking as the completeness of the generation was a key design of this investigation, and, due to the rapid growth of chemical space the minimisation of the time taken to generate the structures is essential.

Table S3: Benchmarking data for four force fields available in openbabel 3D structure generation tool.

Force Field (FF)	Time to generate C6 dataset / hours /mins	Zero Coord or 'nan' structures	Seconds per structure / s
Universal Force Field (UFF)	0.555 / 33	246	5.69

Generalised Amber Force Field (GAFF)	0.564 / 34	245	5.78
Ghemical Force Field (ghemical)	0.663 / 40	238	6.80
Merck Molecular Force Field (MMFF94)	0.524 / 31	237	5.37

1.2 Generation of Unsaturated Hydrocarbons

Unsaturations were then introduced into the dataset with an iterative process. This results in the generation of simple alkenes and alkynes, as well as allenes and cumulene functionalities. Furthermore, ring unsaturations were also generated in this process, structures that had been excluded in previously investigations.

The process for introducing unsaturations into the existing hydrocarbon dataset was performed using a purpose written script. The saturated structures were converted to 3D RDKit molecule objects and each bond examined iteratively. Bonds that could be unsaturated were identified as those matching the SMARTS pattern "[#1]~[#6]~[#6]~[#1]", that is, bonds between two C atoms with a least one H atom bound to each of the carbons. A new RDKit molecule object is then created, identical to the first, from which the identified H atoms are removed, and the bond order between the two Cs is increased by 1, i.e: from single to double, or double to triple.

As for the generation of the saturated structures, InChI strings are generated and used to both minimise generation of duplicates and to allow for diastereoisomer generation, here E-/Z-double bond isomers, rather than point diastereoisomers. Exclusion of duplicates in the generation was more prevalent in this process than in the previous generation. This is because simple, symmetrical structures, such as ^{n}propane, would generate many (in this case, 3) singly unsaturated isomers. However, the base unsaturation process does not differentiate between generation of ^{n}prop-1-ene, ^{n}pro-2-ene and ^{n}prop-3-ene regarding them all as unique. Again, implementation of InChI strings as a checking metric to prevent duplicate generation.

Initially this process produces simple structures with only one new double bond. This process is then repeated, where newly generated unsaturated structures are resubmitted and used to generate structures with additional unsaturations, such as allenes, alkynes, and structures with two non-continuous double bonds. This resubmission process is repeated until no new unsaturated structures are generated within a loop, demonstrating that there are no addition unsaturations possible.

As the unsaturated structures are generated from the existing, fully enumerated, saturated structures, point diastereoisomers already exist within the dataset, and were unsaturated alongside all other structures, preventing need for regeneration. InChI strings also include a stereochemical layer to describe E-/Z-bonding in double bonds (Table S3). Where this information is generated, the same diastereoisomer generation process is used as for saturated structures, through manipulation of the chiral layer of the InChI string.

Table S4: The InChI strings for 2-butene with the different stereochemical layers describe the geometry around the double bond.

InChI=1S/C4H8/c1-3-4-2/h3-4H,1-2H3/b4-3?	
Chiral Tag	2D Geometry
/b4-3+	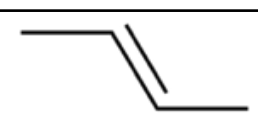
/b4-3-	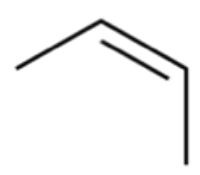

InChI strings do not generate this stereochemistry layer for small rings, defined as fewer than 8 atoms. Hence, a new method was developed. This method involved passing the 3D molecule objects through a series of force fields, for which the same MMFF94 method was used. These force-field calculations were applied with a constraint applied across the double bond, restricting its geometry, the dihedral angles between any substituent groups and at the angles at the unsaturated ring carbons. The process is repeated on the original input molecule up as many times as needed to generate all possible combinations of E- and Z- double bonds in the system.

Due to the limitations of InChI strings not generating stereochemical tags for these structures, duplicates by symmetry cannot be removed easily, and due to the large-scale nature of this dataset manual searching is not feasible. Hence while all care has been taken to remove many duplicates, some persist in the final dataset. These are primarily located in the double-bonds in ring substructure space, and represent no more than 1% of CHX8-enum, or 1.5% of the optimised CHX8.

1.3 Filtering, elimination, and lost Structures.

Around 25% of structures generated for CHX8-enum were removed before optimisation, all either being duplicates or due to failure to generate a 3D structure. None were excluded before this process and their number was not counted towards the CHX8-enum total. Of this removed space, over 95% came from C8 and duplicate structures. Many of these resulted from the diastereoisomer generation process. All molecules that failed to convert to 3D coordinates, returned either a 2D structure, no coordinates or 'nan' coordinates.

The InChI strings of the structures that did not generate 3D structures were examined. As with the duplicates, many resulted during generation of diastereoisomers. This occurred for some structures such as SI5, which do not have diastereoisomers (due to symmetry or structure) however, they are still generated due to inclusion of chiral information in their InChI string.

Table S5: The InChI string for cubane with the different stereochemical layers that arise in generation of InChI strings from 3D structures.

InChI=1S/C8H8/c1-2-5-3(1)7-4(1)6(2)8(5)7/h1-8H/t1-,2+,3-,4+,5+,6-,7+,8-	
Common Name	3D Geometry
Cubane	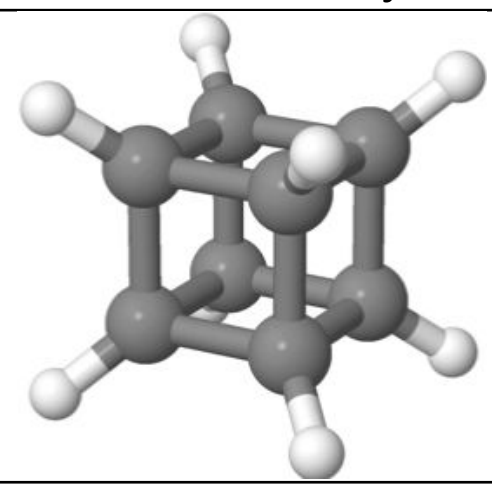

Additionally, there are many unconventional structures that generate some, but not all 3D diastereoisomers. This is highlighted in structures SI6 and SI7. While, the structure does have other chiral centres that would allow it to exhibit further stereochemistry, through symmetry these are enantiomers. In the enumeration process all possible combinations of the chiral labels were generated. In the case of structures SI6-SI13, highlighted in table S4, this was reduced to the unique set of 8 by conversion to canonical InChIs. Only two of those structures generate a 3D representation, SI6 and SI7.

Visualisation of the structures reveals that SI6 is the only sensible stereoisomer of this structure. SI7 represents an isomer where inversion of stereochemistry has resulted in a C-C bond passing through the centre of the molecule, hereafter referred to as "through-bonds". From optimisation of smaller hydrocarbons has been shown to not be possible in spaces of this size. The other six isomers for which

InChIs but not 3D structures were generated can also be assigned to structures with similar through-bonds, or structures which, like cubane, are not actually chiral, despite their assignment.

Table S6: Example structure, highlighting the fallibility of generating diastereoisomers solely through manipulation of the chiral layer of InChI strings.

InChI=1S/C8H6/c1-2-3-6-4-7(3)5(6)8(4,6)7/h2-5H,1H2/t3?,4?,5?,6?,7?,8?	
Chiral Tag	3D Structure
/t3-,4-,5+,6-,7-,8+	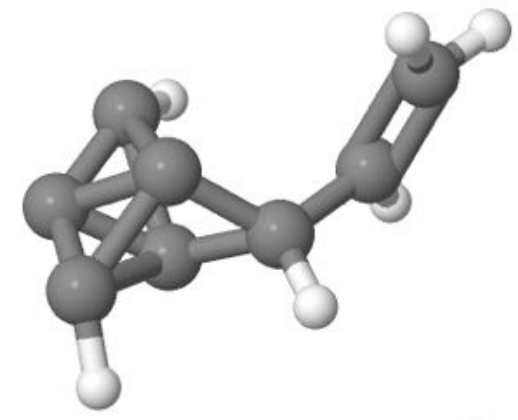
/t3-,4-,5+,6+,7+,8+	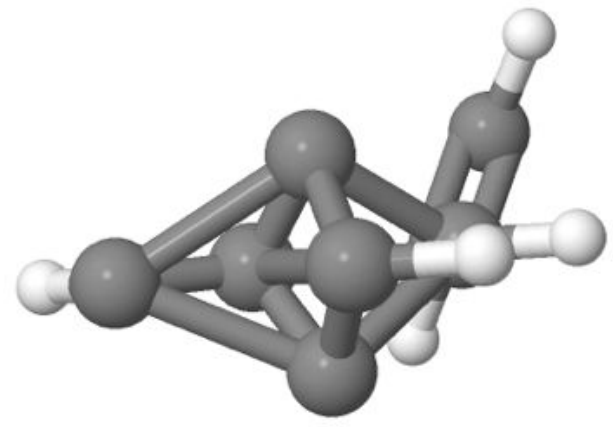
/t3-,4-,5-,6+,7-,8+	N/A
/t3-,4-,5-,6-,7-,8+	N/A
/t3+,4-,5-,6+,7-,8+	N/A
/t3-,4-,5+,6-,7+,8+	N/A
/t3-,4-,5+,6+,7-,8?	N/A
/t3-,4+,5+,6-,7+,8?	N/A

This, alongside the previously mentioned duplication of both point and double-bond diastereoisomers, is responsible for the molecules for which 3D structures were not generated in this enumeration. However, as discussed here, their omission does not result in the loss of any hydrocarbon skeletons, novel or otherwise and therefore is inconsequential.

2. Optimisation and Separation of CHX8

Throughout this investigation calculations were performed using DFT with the PBE0 functional, D4 dispersion correction and def2-TZVP basis sets in the gas phase.[6–10] The calculations were run using Orca 5.0.4 software.[11,12] Structure visualisation was performed using Jmol 14.32, Avogadro 1.99 and Maestro 2023-3.[13–15]

2.1 General Details of Optimisation

Once generated, the structures were optimised using density functional theory (DFT) with the PBE0 functional with D4 correction and def2-TZVP basis set. Largely this was a simple process with 66.5% (51,548) of the structures converging to a ground state structure with no imaginary frequencies after one optimisation process. The remaining 20,000 structures largely converged to this same imaginary-frequency free ground state within one or two additional optimisation processes.

2.2 Post-Optimisation Processing

During the optimisation process, the structures undergo conformational changes as the positions of the atoms are moved to reduce the energy of the structure into that of a local minimum. In most cases this corresponds to the optimised geometry of a given structure. However, in some cases significant structural rearrangement occurs, for example when the input structures were highly strained. This resulted in the optimised, output molecules being different to the input molecules (Figure S2).

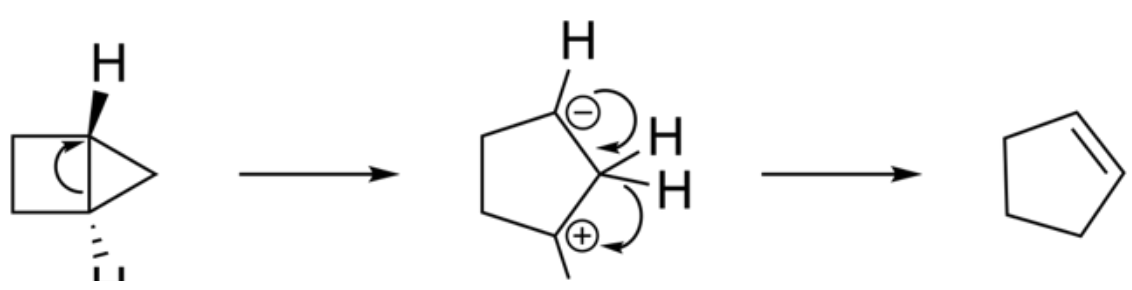

Figure S2: Rearrangement of bicyclo[2.1.0]pentane to E-cyclopentene via ring-opening and hydride migration. The rearrangemnet in this manner is observed in the DFT optimisation.

The final CHX-enum dataset consists of all 77,523 input structures and their related output geometries. This represents all mathematically feasible hydrocarbons with up to eight carbon atoms that have been generated in this study. As the structures that did not rearrange during the optimisation process represent those that could, in theory, be accessed synthetically, a subset of CHX-enum, CHX8 was created containing only those structures that were stable to, and did not rearrange upon optimisation.

Structures that were rearranged were identified through comparison of the InChI strings describing the input and output structures. Input InChIs were taken either from the initial structure generation process or were regenerated from the .mol or .sdf files. Output InChIs were generated using OpenBabel's "OBConversion" package. A script was produced that rebuilt OBMol objects using the atom type, coordinates, and bond order information contained within the Orca frequency calculation output, before conversion to InChI strings that reflect the bonding and connectivity in the products.

Consequently, this process highlights 'rearranged' structures as only those for which the core atom to atom bonding has changed. InChI strings do not contain and information about the type of bond and so structures which have the same bonding but have reduced bond orders are not highlighted as different.

The rearranged structures were analysed to ensure that they did not identify or represent any hydrocarbon skeletons missing from the dataset. It was demonstrated that all structures rearranged though either pericyclic or charge-transfer type reaction mechanisms, resulting in zwitterionic, carbene, or closed-shell structures.

All closed-shell structures were shown to be contained within CHX8. Skeletal equivalents of the zwitterionic and carbene structures, that is structures with the same core carbon backbone but different H atom counts were also found in CHX8. Finally, to ensure that known structures of interest had not been found to rearrange in this investigation, the PUG-REST interface from PubChem was used to probe the presence of these structures within that databased. Out of the 46,027 rearranging structures in CHX8-enum, only 102 structures were identified on this search. Of the rearranging structures that were identified in PubChem, 75 had no literature references at all and were only mentioned in patent documentation. Investigation of the PubChem match and the literature surrounding the remaining 27 structures demonstrated RDKit excluding stereochemical information during the canonicalization before searching none of which were found when stereochemistry was correctly specified, 8 structures had been discussed as curiosities, but remain unsynthesized or exist as non-ground state structures.
The dataset has been uploaded to the Nottingham Repository (http://doi.org/10.17639/nott.7626) and contains all optimised 3D structures, as well as optimisation and frequency calculation output files.

3. Navigating the Dataset

3.1 File contents and hierarchy

CHX8 has been divided into three internal datasets for ease of navigation. These are CHX7, composed of all structures with up to 7 heavy atoms; CHX8-sat, with only saturated eight-carbon hydrocarbons; and CHX-8unsat, containing the unsaturated eight-carbon structures. In addition, all analysis files collected from the dataset is included.

Within each of the three sub-datasets, there are four collections of files, alongside the analysis specifically performed on that sub-dataset. These are finished_mol, with the input geometries used for the calculations; finished_opt, containing the last .out file from optimisation calculations; finished_xyz, with the final, optimised xyz geometry for each structure; and finished_freq, containing all the frequency output files for each structure. (Figure S3)

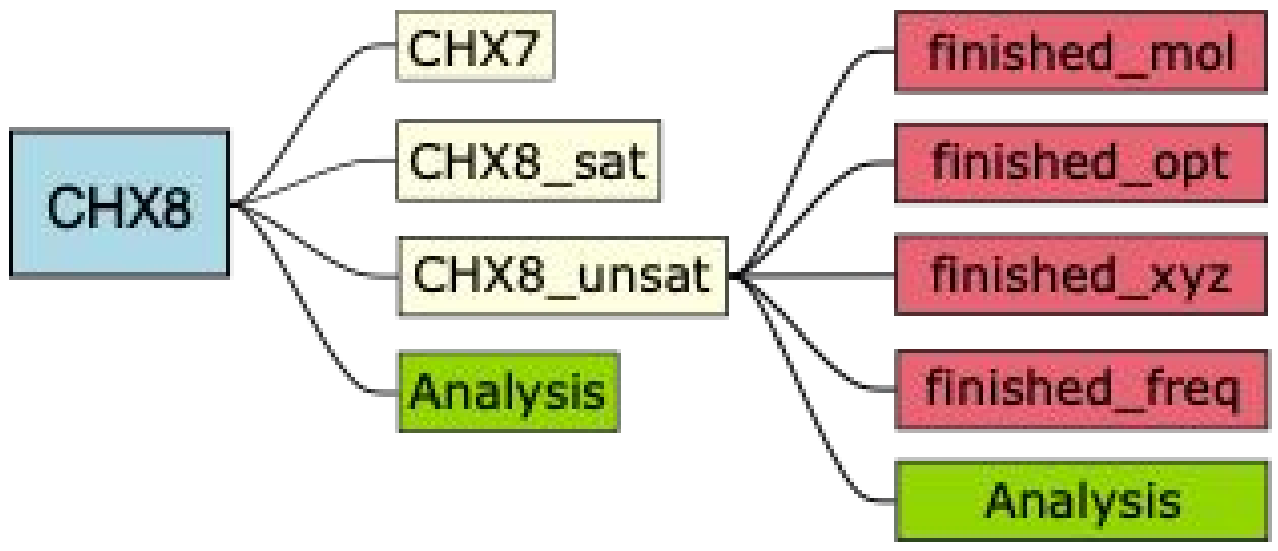

Figure S3: Graphical representation of the file layout of CHX8.

When searching the dataset for a specific structure it is suggested to look for the SMILES or InChi of the desired molecule within the input_inchi.txt or output_inchi.txt files. (S3.3)

3.2 Identifying geometries before and after optimisation

Custom python scripts were written to generate a list of InChIs describing each input and output molecule, these are then stored in the input_inchi.txt and output_inchi.txt. The InChI strings contained within both files are then compared for any changes in connectivity. Structures with differing InChI strings have undergone structure changes during the DFT optimisation and hence are removed from the core dataset.

3.3 Structure searching within the dataset.

Searching for a specific structure within the dataset is made trivial through the inclusion of these input and output_inchi.txt files. Both files are laid out in the same way:

Filename	SMILES	InChI
Butanesat1	CC(C)C	InChI=1S/C4H10/c1-4(2)3/h4H,1-3H3

Where the file name, is denoted by a fixed prefix describing the number of carbons and whether it is saturated or unsaturated, as shown in table S7.

Table 7: The prefixes used throughout the CHX dataset to describe each hydrocarbon.

Number of Cs	Saturated Prefix	Unsaturated Prefix
1	methanesat	--
2	ethanesat	ethaneunsat
3	propanesat	propaneunsat

4	butanesat	butaneunsat
5	pentanesat	pentaneunsat
6	hexanesat	hexaneunsat
7	heptanesat	heptaneunsat
8	octanesat	octaneunsat

This is followed by an integer number which denotes the order of structure of generation. As a result each molecule has a unique filename within the dataset, for example hexaneunsat513 denotes benzene.

Additionally, within CHX8_unsat generation of double bonds in rings was automated, the *cis*- and *trans*-isomers of these structures are named octaneunsatXXXc.mol or octaneunsatXXXt.mol, where XXX is the structure number. In systems where there are multiple double bonds there were enumerated iteratively, giving: octaneunsatXXXcc.mol, octaneunsatXXXct.mol, octaneunsatXXXtc.mol, and octaneunsatXXXtt.mol, which occurs in the same manner for systems with three or four double bonds in rings.

4. Strain and Relative Strain Energy Calculation

Strain is a fundamental and important concept in organic and synthetic chemistry, allowing for the estimation of molecular stability and reactivity.[16–19] It is the excess energy resulting from structural distortions and steric interactions relative to a reference structure considered to have zero strain.[20] Traditional isodesmic reactions[21] rely on the decomposition of each target structures into specific acyclic analogues, this approach is not tractable for large-scale libraries of molecules, such as that presented here. The selection of different reference molecules for each target structure would require significant manual or algorithmic input, which with different references across many molecules would increase time cost and introduce significant noise into the results. Consequently, a generalised expansion of the methodology established by Dudev and Lim is employed.[22] This formulation allows for the calculation of the relative stain energy (RSE) of any hydrocarbon relative to any other structure. In this work cyclohexane is always employed as the zero-strain reference structure to maintain consistency across the dataset.

4.1 The RSE equation.

The following strain equation has been developed and adapted from existing work to allow for its high-throughput use across CHX8. In this work, RSE is defined as the energetic difference between the system of interest (E_n) and a hypothetical strain-free reference (E_r), here cyclohexane is used.

$$\mathrm{RSE} = \mathrm{E_n} - \mathrm{E_r} - (\mathrm{c_n} - \mathrm{c_r})\mathrm{E_{CH_2}} + \mathrm{E_{unsat}} \qquad \text{(S4.1)}$$

This equation effectively constructs a 'virtual' isodesmic reaction for each molecule. By explicitly accounting for differences in carbon count $(\mathrm{c_n} - \mathrm{c_r})$, and degree of unsaturation, $\mathrm{E_{unsat}}$, we ensure that RSE represents only the geometric strain of the system, independent of stoichiometry or functional group density. This provides a consistent, automatable metric that matches the thermodynamic rigor of isodesmic schemes without introducing additional noise or error introduced by manual or diverse reference selection in a high-throughput analysis.

In Equation S4.1 E_n and E_r are the Gibbs energies of the molecule and the reference (cyclohexane), respectively. The $(\mathrm{c_n} - \mathrm{c_r})\mathrm{E_{CH_2}}$ term functions as a stoichiometric correction, while $\mathrm{E_{unsat}}$ acts as a function group correction. Together, these two terms 'morph' the reference cyclohexane into a strain-free version of the target.

Within the $(\mathrm{c_n} - \mathrm{c_r})\mathrm{E_{CH_2}}$ term, $\mathrm{c_n}$ and $\mathrm{c_r}$ are the number of C atoms present in the structure of interest and the reference structure respectively. The $\mathrm{E_{CH_2}}$term describes only the energy difference resulting from the addition of a new, unstrained, CH_2 unit into an existing hydrocarbon. It was found from the average energy difference between each of the linear hydrocarbons contained within the dataset. (Equation S4.2)

$$\sum \mathrm{E_{CH_2}} = \sum_{\mathrm{m=2,}}^{\mathrm{m=7}} \mathrm{E_{C_{m+1}H_{2(m+1)+2}}} - \mathrm{E_{C_mH_{2m+2}}}$$

$$\mathrm{E_{CH_2}} = \frac{\sum \mathrm{E_{CH_2}}}{6} \qquad \text{(S4.2)}$$

$\mathrm{E_{unsat}}$ is composed of three additional terms (Equation S4.3). Each term in $\mathrm{E_{unsat}}$, describes the energy difference associated with introducing a prototypical unsaturation, rings, double, and triple-bonds into the system.

$$\mathrm{E_{unsat}} = (\mathrm{r_n} - \mathrm{r_r})\mathrm{E_{ring}} + (\mathrm{d_n} - \mathrm{d_r})\mathrm{E_{double}} + (\mathrm{t_n} - \mathrm{t_r})\mathrm{E_{triple}} \qquad \text{(S4.3)}$$

$\mathrm{E_{double}}$ and $\mathrm{E_{triple}}$ are derived by introducing a double or triple bond is introduced between the second and third C atoms of linear, unbranched hydrocarbons (*n*-butane to *n*-octane). Initially, this was performed using the same principle but with terminal double and triple bonds. As highlighted in section 4. 2 internal unsaturation were employed over terminal ones to avoid the systematic underestimation of RSE values.

$\mathrm{E_{ring}}$ is more complicated than the other terms. Rather than finding the average energetic cost of introducing a C-C single bond into a linear system to form a ring, the same approach is used as presented in the paper by Dudev and Lim.[22] Homolytic bond fission energies were used to account for the specific loss of two C-H bonds upon formation of a C-C cycle (Equation S4.4-S4.6).

$$E_{ring} = 2E_{C-H} - E_{C-C} \quad \text{(S4.4)}$$
$$E_{C-H} = E_{ethane} - E_{ethyl\ radical} \quad \text{(S4.5)}$$
$$E_{C-C} = E_{ethane} - 2E_{methyl\ radical} \quad \text{(S4.6)}$$

A similar approach to that employed here is presented by Coia *et al.*[19] but it is noted that the method used here allows for comparison without recalculation of the correction parameters for each molecule and additionally allows for different degrees of unsaturations from both rings and multiple bonds to be accounted for. By treating each type of unsaturation independently, electronic stabilisation and steric distortions from the expected baselines can be identified.

4.2 Benchmarking of the RSE calculation approach

The approach for calculating RSE employed here allows for comparison of any two structures, regardless of how related they might be. This allows for a general universal strain scale to be created, with the RSE of all structures, relative to a given zero-point standard created. Here the reference point is cyclohexane, chosen as it is often considered to be a zero-strain ring system.

Before complete implementation of the RSE calculation approach, it was important to ensure the method was robust. The final method arose from three iterative steps. Method 1 treated E_{unsat} as a single term, using the same correction value for ring, double and triple bond unsaturations. However, this resulted in significant overestimation of RSE for double-bond containing structures.

Method 2 treats each unsaturation with a separate term, however terminal alkene and alkyne units were used to derive the correction terms, resulting in underestimation of RSE. Finally, method 3 was adapted calculating the correction terms from alkene and alkyne functionalities between the second and third C atoms within linear chains. The RSEs calculated with these three methods for a range of structures are presented in table S8.

Table S8: Strain energies for structures from benchmarking and validate the RSE calculation approach used here. All values are relative to cyclohexane, unless otherwise stated.

Structure	Literature	Method 1	Method 2	Method 3
cyclopropane[a]	27.5	27.1	27.1	27.1
Cyclopropene[a]	55.2	70.6	51.4	55.0
Cyclobutane[a]	26.5	25.5	25.5	25.5
Cyclobutene[a]	28.4	46.0	26.8	30.4
cyclopentane[a]	6.2	5.2	5.2	5.2
cyclopentene[b]	4.7	22.5	1.3	4.9
Z-cyclohexene[b]	0.7	15.8	-3.4	0.3
Z-cycloheptene[a]	3.6	19.4	0.2	3.8
E-cycloheptene	23.2	67.6	32.7	29.0
Z-cyclooctene[a]	4.2	--*	2.2	5.9
E -cyclooctene[a]	16.4	--*	18.1	21.8
cis-bicyclo[1.1.0]butane[a]	63.9	58.0	58.0	58.0
cis-bicyclo[5.1.0]octane[a]	27.9	25.2	25.2	25.2
trans-bicyclo[4.1.0]octane[a]	40	41.9	41.9	41.9
bicyclo[1.1.1]pentane[a]	68	59.1	59.1	59.1
Bicyclo[2.1.1]hexane[a]	37	30.2	30.2	30.2
bicyclo[2.1.1]hex-2-ene[a]	51	60.5	41.3	44.9
bicyclo[2.2.1]hept-2-ene[a]	19.2	29.4	10.2	13.9

[a]See ref [18], [b]See ref [23] * Optimised structures were not available during testing of this model.

To ensure that the RSE gave reasonable, logical results, the increase of RSE as a function of general molecular formula, or by proxy, degree of unsaturation, was considered. As structures become more unsaturated, either due to an increasing number of rings or the presence of multiple bonds, the RSE increases as the structure is forced to distort away from their ideal, purely tetrahedral geometry (Figure

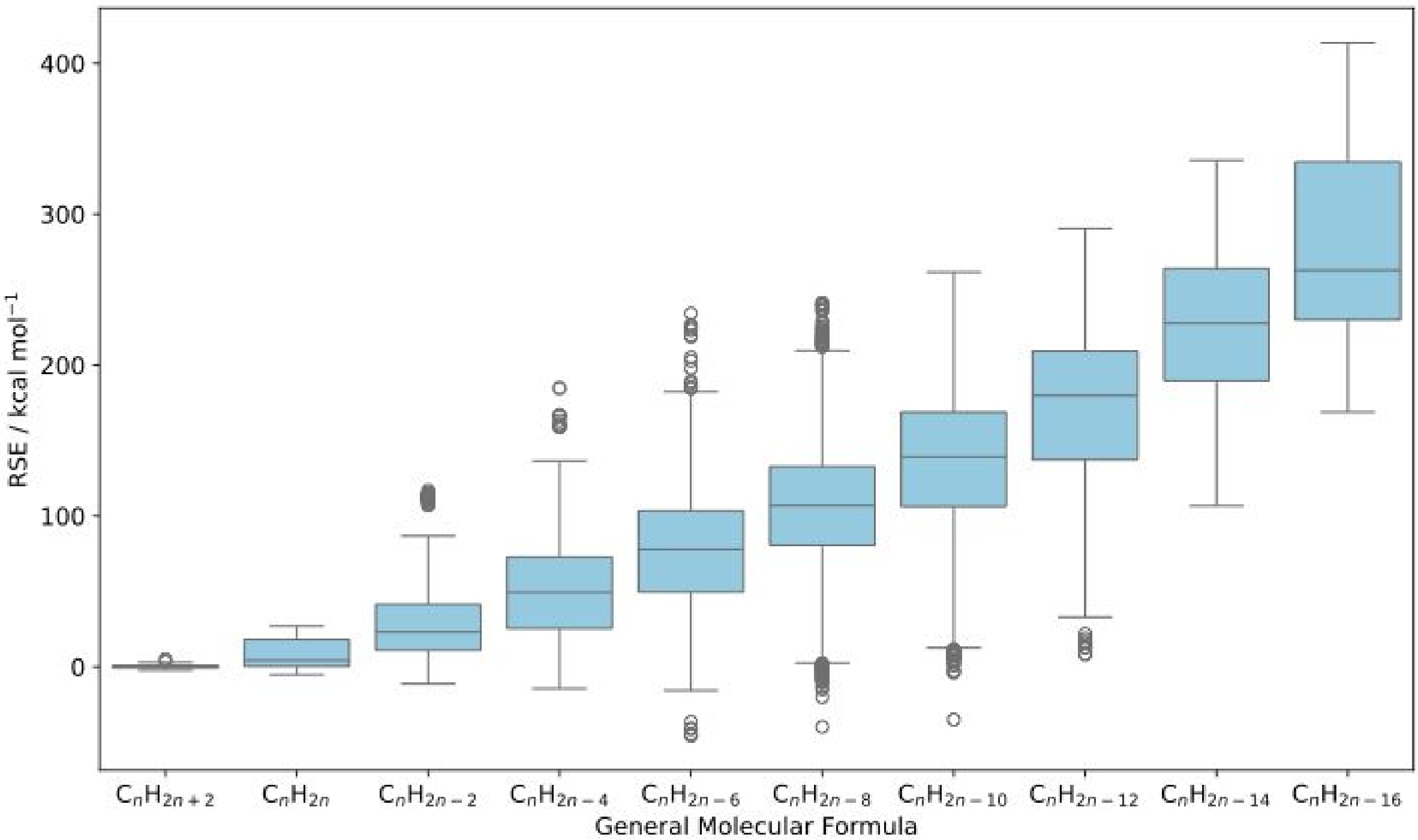

Figure S4: RSE of the structures contained within CHX8 as a function of generalised molecular formula.

S4).

Within figure S4, structure with the general formula C_nH_{2n-6} demonstrate the widest range of RSEs. This is primarily driven through additional structures with RSEs below -10 kcal mol^{-1}. These low energy structures arise with the emergence of aromaticity that becomes possible with four unsaturations (three double bonds and a ring). As additional unsaturations are introduced fewer structures are seen that benefit from this the energetic stabilisation arising from aromaticity. In CHX8 there are 6 possible phenyl containing molecules, which include three structural isomers of xylene, toluene, ethylbenzene and benzene itself. Only ethylbenzene may be further unsaturated to give additional structures, as styrene, or as ethynylbenzene, each of which may be identified as the lowest energy structures in C_nH_{2n-8} and C_nH_{2n-10} respectively in figure S3.

4.3 The Effect of Me-Substitution on the RSE of Small Rings.

To investigate the substituent effects on the RSE of standard hydrocarbon backbones the effect of introduction methyl groups onto the small-ring systems was investigated. In small ring systems, there is a careful balance between destabilisation from steric interactions and RSE reduction from hyperconjugation upon introduction of substituent groups. When only one methyl group is introduced onto the simple ring systems present in this dataset an average reduction in RSE of 2.7 kcal mol^{-1} is observed across all structures. These results are highlighted in table S9.

Table S9: RSE for small to medium ring systems with and without methylation at one position on the ring. All RSE values are given in kcal mol^{-1}.

Ring System	Simple Ring	Methylated Ring	ΔRSE
cyclopropane	27.1	24.2	-2.9
cyclobutane	25.5	21.9	-3.5
cyclopentane	5.2	2.9	-2.3

Ring System	Simple Ring	Methylated Ring	∆RSE
cyclohexane	0.0	-2.7	-2.7
cycloheptane	5.7	3.8	-1.9

Although the ∆RSE upon inclusion of a methyl group in these instances is small, it highlights the ability of substituents to stabilise strained and potentially disfavoured hydrocarbon backbones *via* electronic effects. This demonstrates that while simple rings are the focus here, more complex substituents, such as bulky groups or π-donors, could be used to thermodynamically favour highly strained geometries that might otherwise be transient. Further investigation into the substituent effects of neighbouring π-systems is considered in SI 6.2.2, demonstrating more pronounced stabilisation effects in specific electronic environments.

5. Comparison to other Databases/Datasets

5.2 Comparison to VQM24

VQM24 is a recent exploration of all possible structures for the molecular formulae of C_xH_y, for x = 1-5 and y = 0-12. This chemical space uses molecular formulae as a starting point, using the SURGE enumeration software.[24] Through the inclusion of non-carbon heavy atoms, VQM24 generates 258,000 molecules. Of this only 135 are hydrocarbons, and only 123 of those represent closed-shell isomers. In both occurrences, CHX8 contains 154 $C_{1\text{-}5}H_{0\text{-}12}$ exhibiting a region of chemical space 14-25% larger than that described by VQM24.

Many of the structures not found in VQM24 are diastereoisomers, both point and double-bond, as these are not generated by the SURGE process. However, it was also noticed that some fundamental structures were missing from the dataset, including ethane, although ethene and ethyne were present.

5.3 Comparison to PubChem

PubChem represents one of the largest publicly available datasets, detailing over 110 million structures. Determination of which structures are detailed within PubChem provides a practical demonstration of how well explored hydrocarbon chemical space has been up to this point. Exploration of PubChem was limited only to the CHX7 dataset to ensure that data collection could be completed on a reasonable timescale.

Searching in PubChem was performed through interaction with the Power User Gateway-Representational State Transfer (PUG-REST) interface. The SMILES string for each structure was searched for within PubChem. Initially, stereochemical information was included in the search and if a negative result was returned, the stereochemistry was removed and the search performed again.

The initial search, revealed that only 44% of CHX8 was reported in any capacity within PubChem. This decreased to less than 8% when stereochemistry was included within the search criteria.

5.4 Comparison to Reaxys

The REAXYS dataset also details a large array of known chemical space with more than 31 million commercially available substances reported within it. Through application of the REAXYS filtering processes (see screenshot), the SMILES strings for all structures that occupied the same region of chemical space as CHX7 were extracted. Only CHX7 was chosen as the filtering system on REAXYS is not infallible and increasing to the CHX8 chemical space would introduce additional errors. One example of such errors was upon the inclusion of a filter that would remove structures with the ‘radical’ tag within the REAXYS dataset, which was found to remove structures like ^{n}heptane.

The list of SMILES downloaded from REAXYS therefore required a degree of post-processing to remove structures such as carbenes, radicals and deuterated structures, among others. After post-processing, there were 1328 unique hydrocarbons, of which 1315 were found to be present in CHX7. The remaining 13 structures were found in the wider CHX-enum dataset. CHX7 therefore contains more than twice the number of structures REAXYS does for the same region of chemical space.

The advantage of investigating REAXYS as well as PubChem is the large number of commercially available structures listed on REAXYS. This filter was then additionally applied to the REAXYS dataset, reducing it to only 703 hydrocarbons, all of which were present in CHX7. However, again it is known that this value is not correct and the true value is likely to be lower. Some structures on REAXYS, such as tetrahedrane, are listed as commercially available even though they have yet to be achieved synthetically.

6. Further Discussion from the Main Text

6.1 Alkynes and Allenes in Rings

6.1.1 On the stability of small-ring allenes and alkynes

The smallest cyclic allenes, **S4** and **S6**, are known to possess complex potential energy surface (PES), which due to the high H:C ratio contains a large number of biradical and carbene structures.[25–27] Cyclopropadiene, **S4**, is known to exist as the diradical species cyclopropenylidene, **S5**, which has been spectroscopically detected in interstellar media and on Saturn's Moon Titan.[26,28] Hence, its stability here is unsurprising (Figure S5).

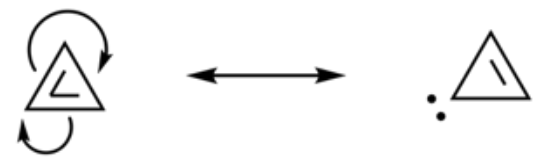

S4 70.9 **S5**

*Figure S5: The resonance of cyclopropa-1,2-diene, **S4**, to give the cycloprop-1-enyl-2-idene, **S6**. RSE is given in kcal mol^{-1}, and is only given once, calculated for a **S5**-like structure.*

In CHX8 singlet cyclobuta-1,2-diene, **S6**, is unstable to optimisation and freely rearranges to **S7**, bicyclo[1.1.0]butylidene (Figure S6). The triplet structure is found to be stable to optimisation and so was used to generate a RSE value moving forward. Contrary to this, previous studies on the C_4H_4 PES have found the singlet structure to be stable, albeit as the diradical, carbene species, **S8**, alongside identification of a transition state, and associated energy barrier of 8.9 kcal mol^{-1} for this rearrangement.[25]

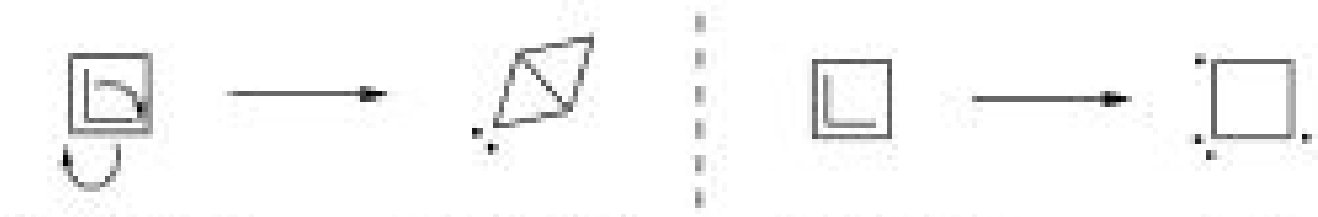

S6 89.2* **S7** 76.8 **S6** 89.2* **S8**

*Figure S6: The electrocyclisation of singlet cyclobuta-1,2-diene, **S6**, to give bicyclo[1.1.0]butylidene is also shown. RSE is given in kcal mol^{-1}, and where values calculated from triplet structures are denoted*

This difference in results is attributed to the use of the B3LYP functional for those calculations, in addition to use of CASSCF calculations for validation of any biradial species, whereas here only PBE0-D4/def2-tzvp level is used. The larger cycles have all been found and identified in previous experiments, either as transient intermediates or through trapping experiments.[29–31]

6.1.2 On the apparent stability of cyclopropyne

Within this investigation a ground state structure with no imaginary frequencies has been identified for alkyne-23, cyclopropyne. The structure is an asymmetrical, planar molecule retaining the same carbon backbone as the input structure. Previous computational studies on the synthetically hypothetical cyclopropyne all find the singlet structure to be, by some order, a saddle point.[32–34] The traditional, expected cyclopropyne structure with a tetrahedral sp^3 C atom bearing the two H atoms, tetrahedral cyclopropyne, is often found to be a second order saddle point. The two associated imaginary frequencies are consistent across a range levels of theory and correspond to: A rotation of the CH_2 group towards

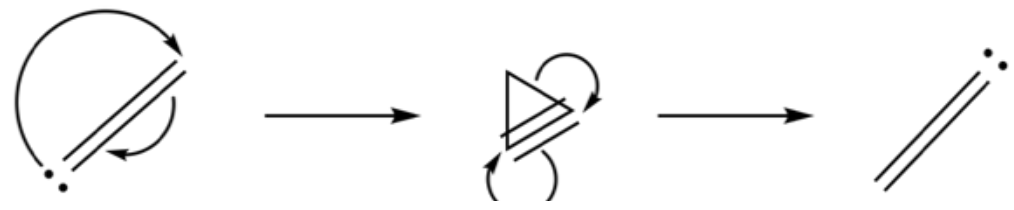

Figure S7: The automerisation of propa-dienyl-carbene via cyclopropyne

planarity, and an asymmetrical stretching of a C-C bond. Other studies have considered a symmetrical, planar cyclopropyne similar to that observed in CHX8, finding it to be a transition state structure with only one imaginary frequency, corresponding to the asymmetric C-C stretch. The general consensus of previous computation studies surrounding the singlet cyclopropyne structure is that it represents some saddle point in the automerisation of propadienylcarbene (Figure S7)
Here, triplet tetrahedral cyclopropyne is also found to be a first order saddle point, with one imaginary frequency corresponding to the asymmetric C-C bond stretch, while the triplet planar asymmetric structure rearranges during the optimisation to give dienylcarbene.

As the ground state, singlet structure does not fit with existing computational studies of the structure, some computational benchmarking was performed. This determined that this ground state structure was an artefact of the functional employed here and appears to be independent of the basis set used. The results of this benchmarking are summarised in table S10.

Table S10: Functionals and basis sets with which the two structures of cyclopropyne was optimised. These are presented alongside a binary result of whether the structure did not rearrange significantly in optimisation, to give a ground state structure with no imaginary frequencies.

Functional	Basis Set	Did the tetrahedral structure optimise with the same connectivity to a ground state?	Did the planar, asymmetric structure optimise with the same connectivity to a ground state?
PBE0	def2-TZVP	No	Yes
PBE0	6-31G*	No	Yes
PBE	6-31G*	No	No
PBE	def2-TZVP	No	No
B3LYP	6-31G*	No	No
B3LYP	def2-TZVP	No	No
ωB97M-V	def2-QZVPP	No	No
ωB97M-V	aug-cc-pVQZ	No	No

6.2 On the stability of anti-Bredt structures

6.2.1 Correlation between S value and RSE.

It was demonstrated that Fawcett's S value, that is the number on non-bridgehead ring atoms in an anti-Bredt structure, was a poor indicator of the RSE of a molecule. This was surprising as structures with higher S values have more non-bridgehead cyclic ring atoms and therefore should have increases flexibility and consequently lower RSEs.

The method for RSE calculation employed here accounts for the strain of the whole molecule, rather than only that of ring sections. As S values increase, there are increasing numbers of positions for substitution of strained groups onto the core skeleton, as well as additionally non-bridgehead ring positions that may be unsaturated. These factors may all give the impression of increasing bridgehead strain in systems with larger S values. This results in the observation that structures with larger S values demonstrate a higher associated range of RSEs (Figure S8). On average, however, both substituted and unsubstituted anti-Bredt skeletons shown reduction in RSE with increasing S, highlighted in Table S11. The reasoning for S=5 demonstrating a lower RSE than S=6 across all anti-Bredt's structures arises from the presence of a higher number of more substituted, singly unsaturated, anti-Bredt's structures within CHX8.

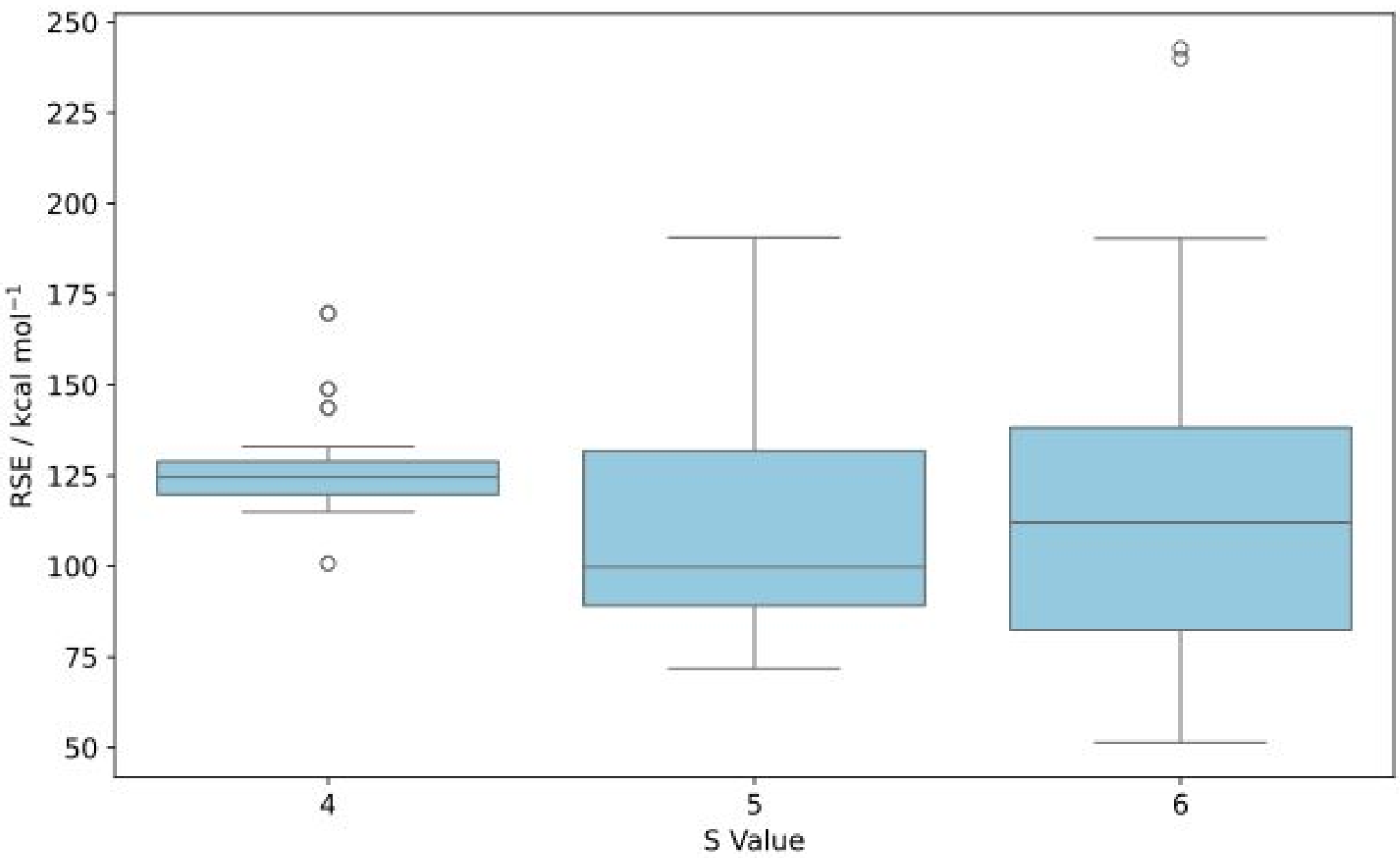

Figure S8: RSE as a function of the Fawcett parameter (the number of non-bridgehead ring atoms) for all anti-Bredt structures in CHX8

Table S11: Average RSE in kcal mol^{-1} for the core anti-Bredt skeleton structures and all anti-Bredt structures contained within CHX8.

	Average RSE / kcal mol^{-1}	
S Value	Core anti-Bredt skeletons	All anti-Bredt structures
4	109.8	126.4
5	81.6	112.9
6	45.1	116.9

6.2.2 Improving the stability of anti-Bredt structures.

While novel approaches for tackling the synthesis of anti-Bredt's structures often focus on accessing the unsubstituted core frameworks, small, highly cyclic hydrocarbons are rarely useful as standalone targets. Their broader value is more likely to lie in their incorporation into functional molecules, for example, as hydrocarbon bioisosteres within drug-like scaffolds.

Therefore while synthetic routes the result in these isolated hydrocarbons are exciting and useful, methods for accessing these highly strained structures with substituents are also beneficial. The most logical approach for the synthesis of anti-Bredt's structures is through reduction in the strain associated with these structures. This is a non-trivial problem, as, while removing the bridging atoms or unsaturations can make them more accessible, this removes the features that makes these structures interesting. Therefore, approaches should look at methods by which the total strain on the unsaturated bridgehead atom can be reduced.

One approach could be through the introduction of resonantly electron-withdrawing functional groups β to the unsaturated bridgehead. These functional groups allow for the double bond to be delocalised away from the bridgehead atom, reducing the strain at this position. This conclusion is supported through studies of Wiberg et al.[35] who, in 1990 successfully trapped a derivative of **S11** with a phenyl group β to the unsaturated bridgehead, **S13**. This structure was generated outside of the enumeration and an RSE was calculated. It was found to have an RSE nearly 70 kcal mol^{-1} lower than that of **S11**. (Figure S9)

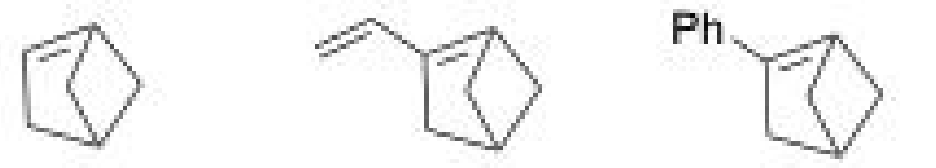

S11 106.4 **S12** 100.8 **S13** 38.6

Figure S9: Anti-Bredt's structures alongside their RSE in kcal mol-1. Increasing the delocalisation ability of the group β to the bridgehead unsaturation significantly reduces RSE, demonstrating the stabilisation of the strained functionality.

In CHX8 there were very few structures with an electron withdrawing substituent in proximity to the unsaturated bridgehead atom in anti-Bredt's structures. However, a similar derivative of **S11** was present, where a vinyl group is situated β to the unsaturated bridgehead atom, **S12**. **S12** had an RSE 6 kcal mol^{-1} lower than that of **S11**, highlighting the potential of electron-withdrawing groups to stabilise anti-Bredt's frameworks. (Figure S9)

The relative changes in RSE seen due to the introduction of these groups demonstrates that resonant delocalisation of the electron density away from the bridgehead atom in anti-Bredt's structures can be used to reduce there RSE and therefore make them more synthetically accessible. It additionally demonstrates that these structures may be stable to inclusion within wider structures and frameworks such as drug molecules.

6.3 Trans-ring junctions

6.3.1 Multiple ring junctions on the same central ring.

Inclusion of multiple rings onto the same central ring gives additional detail about the stability of these structures. Initially, it was seen that trans-ring junctions cannot exist adjacent to each other on a ring. The structures undergo a rearrangement to generate the cis-ring junction, due to the pre-orientation of the electron density in the C-C bonding orbitals. When rings are not adjacent, two or more may be both stabilised in a trans- conformation on the same ring, provided the central ring is of sufficient size.

Two trans-3-MRs may be stabilised on a central 6-MR in either of the two non-adjacent isomers. For two trans-4-MRs a central ring of five or more constituent atoms is required. This results from the additional conformational flexibility present in a 4-MR over that of a 3-MR that allows for the distortion on the central ring to be more distributed onto the substituent rings.

One additional, non CHX8 structure was generated to investigate the effects of substituting 3 rings onto the same central ring. A central 6-MR can have up to three non-adjacent rings appended around in, as in tetracyclo[6.1.0.0$^{(2,4)}$.0$^{(5,7)}$]nonane. Larger structures than this were not considered as they would require at least 12 total atoms to study either three 4-MRs or four 3-MRs.

This investigation found that the central 6-MR present in this structure can support all three 3-MRs in a trans- conformation. Based on the previous studies of one or two trans-ring junctions have demonstrated, 3-MRs are more strained and harder to stabilise than larger rings. Therefore, it is proposed that a central 6-MR will be able to support three 4- or larger-MRs in trans-configurations.

Furthermore, due to the requirement of trans-rings non-adjacent rings and the scaling of the central rings that accommodates this, it is expected that four 3-MRs will be stable on a central 8-MR (pentacyclo[9.1.0.0$^{(2,4)}$.0 $^{(5,7)}$.0$^{(8,10)}$]dodecane) which has additional conformational flexibility relative to a central 6-MR. It is suggested that the increased strain resulting from additional trans-ring substituents cannot increase faster than the additional conformation flexibility gained by increasing size of the central ring supporting them.

6.3.2 Trans-ring junctions – Rules for Synthesis and Stability

As a result of the investigations conducted here three rules are presented for the general discussion and potential synthesis of structures including trans-ring compounds (Figure S10):

1. Any ring junction on a central 4-MR or larger (or a 5-MR if at least one is a 3-MR) may exist cis- or trans- and so should be drawn and discussed with explicit stereochemistry.
2. Two or more rings may exist trans- provided a) They are not adjacent and b) They are on a central 5-MR (or 6-MR if at least one is a 3-MR).
3. Any chain of ring junctions is stable in any combination provided condition 2 is fully met for any three continuous ring junctions.

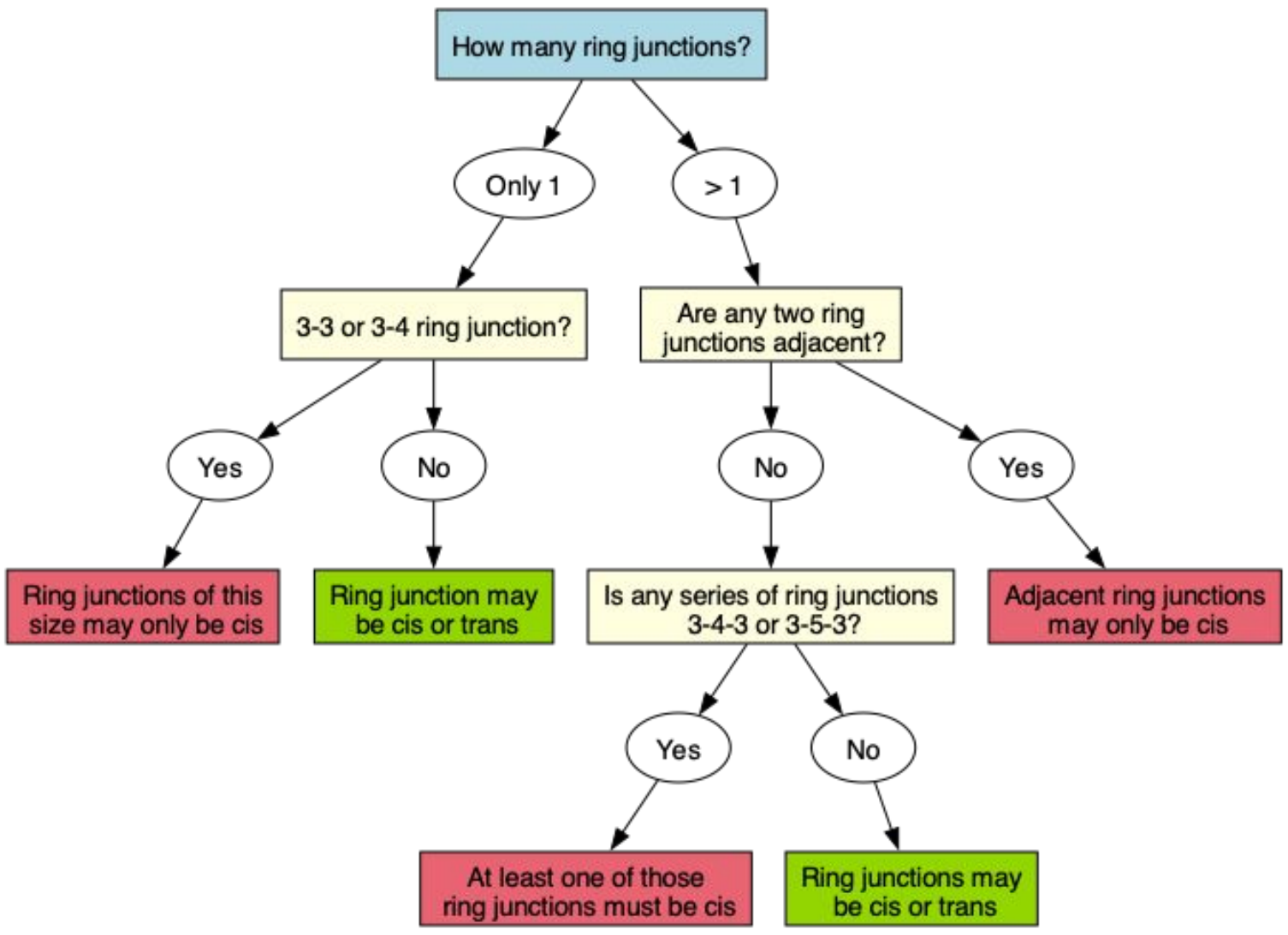

Figure S10: Flowchart showing rules for the inclusion of trans-ring junctions in

7. Structural Diversity in CHX8

7.1 Further Comparison to GDB-13

To quantify the unique chemical space and distribution of structures contained within CHX8, the diversity of hydrocarbon skeletons contained within it were compared to the GDB-13 dataset [36] using Uniform Manifold Approximation and Projection (UMAP) for dimensionality reduction. [37] This was achieved by generating Morgan fingerprints for all molecules in CHX8 and the same region of GDB-13, hydrocarbons with up to 8 C atoms. Fingerprints were generated using RDkit with radius =2 and nBits = 2048. [2] Both datasets were then concatenated into a single data frame to ensure the resultant UMAP plot retained interpretability for a comparison of CHX8 and GDB-13. To reduce noise and computational cost, the data was pre-processed using principal component analysis (PCA)[38,39] reduction with n_components = 50 and random_state = 42. This was then used as an input for the the UMAP reduction, performed with n_neighbors = 30, min_dist = 0.1 and random_state = 42, Figure S11.

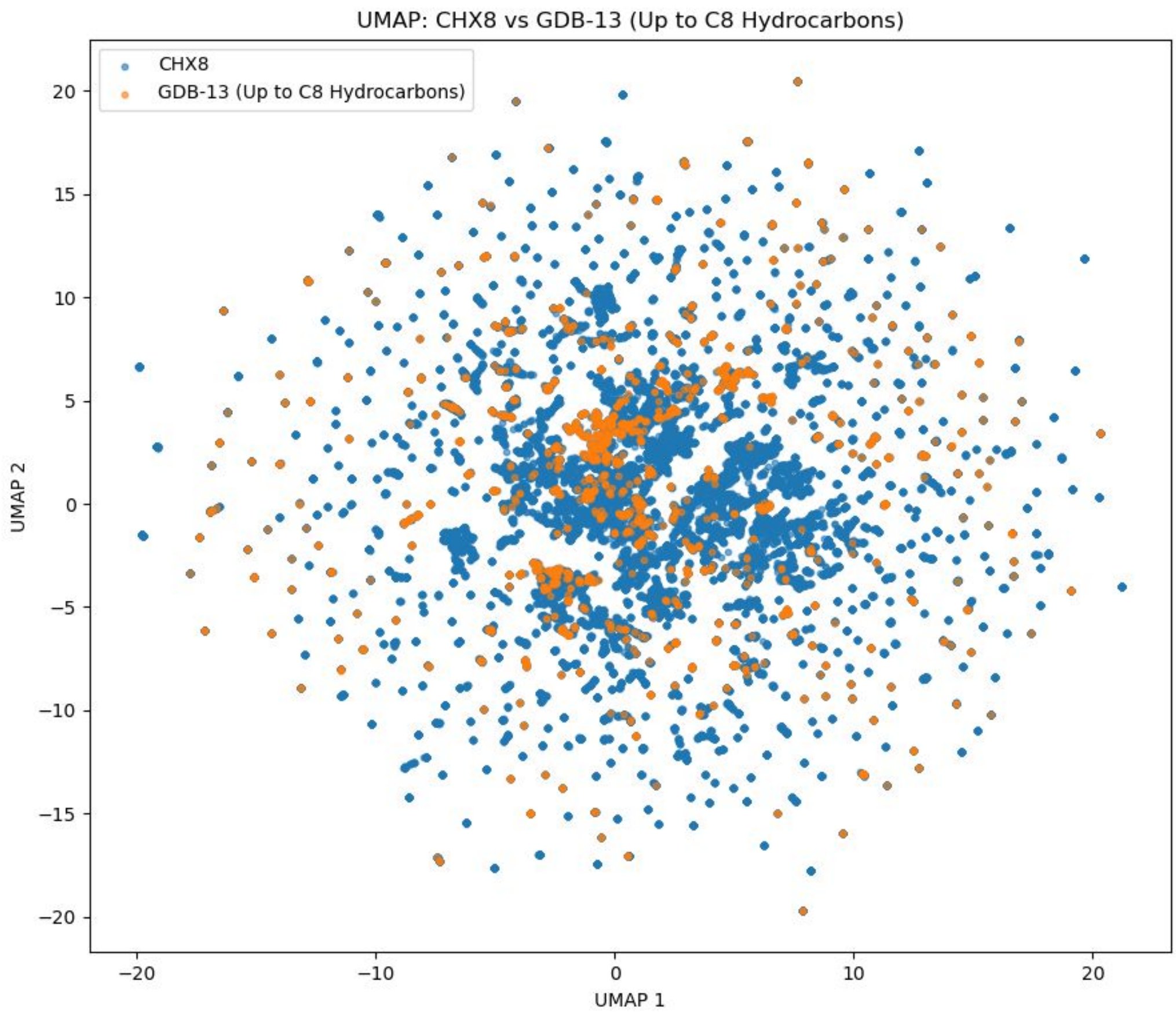

Figure S11: UMAP dimensionality reduction of Morgan fingerprints generated for all molecules in CHX8 and GBD-13. Morgan fingerprints generated with radius =2 and nBits = 2048, data was pre-processed with PCA, n_components = 50 and random_state = 42, before UMAP with neighbors = 30, min_dist = 0.1 and random_state = 42.

The projection reinforces that the additional chemical space explored in CHX8 over that in GDB-13. In figure S11, there is a core cluster containing the largest proportion of GDB-13 structures. Surrounding this core a number of satellite clusters may be identified, which contain few orange, GBD-13, points. These

highlight the hydrocarbon skeletons present in CHX8 that are not in GDB-13, such as the unsaturated small-rings.

7.2 Structure Diversity within CHX8

To gain a better understanding of the structural diversity contained within CHX8 further dimensionality reduction plots were generated. As previously, Morgan fingerprints were generated using RDkit with radius =2 and nBits = 2048, before PCA, UMAP and tSNE plots were generated and the specific details for generating those plots can be found in Figure S12.[37–40]

Across these three plots a colour gradient was applied, where each molecule, or point, was coloured based on its computed RSE value, this provided confirmation that any clusters that were identified within the plots could be interpreted as related structures, and not just random blobs.

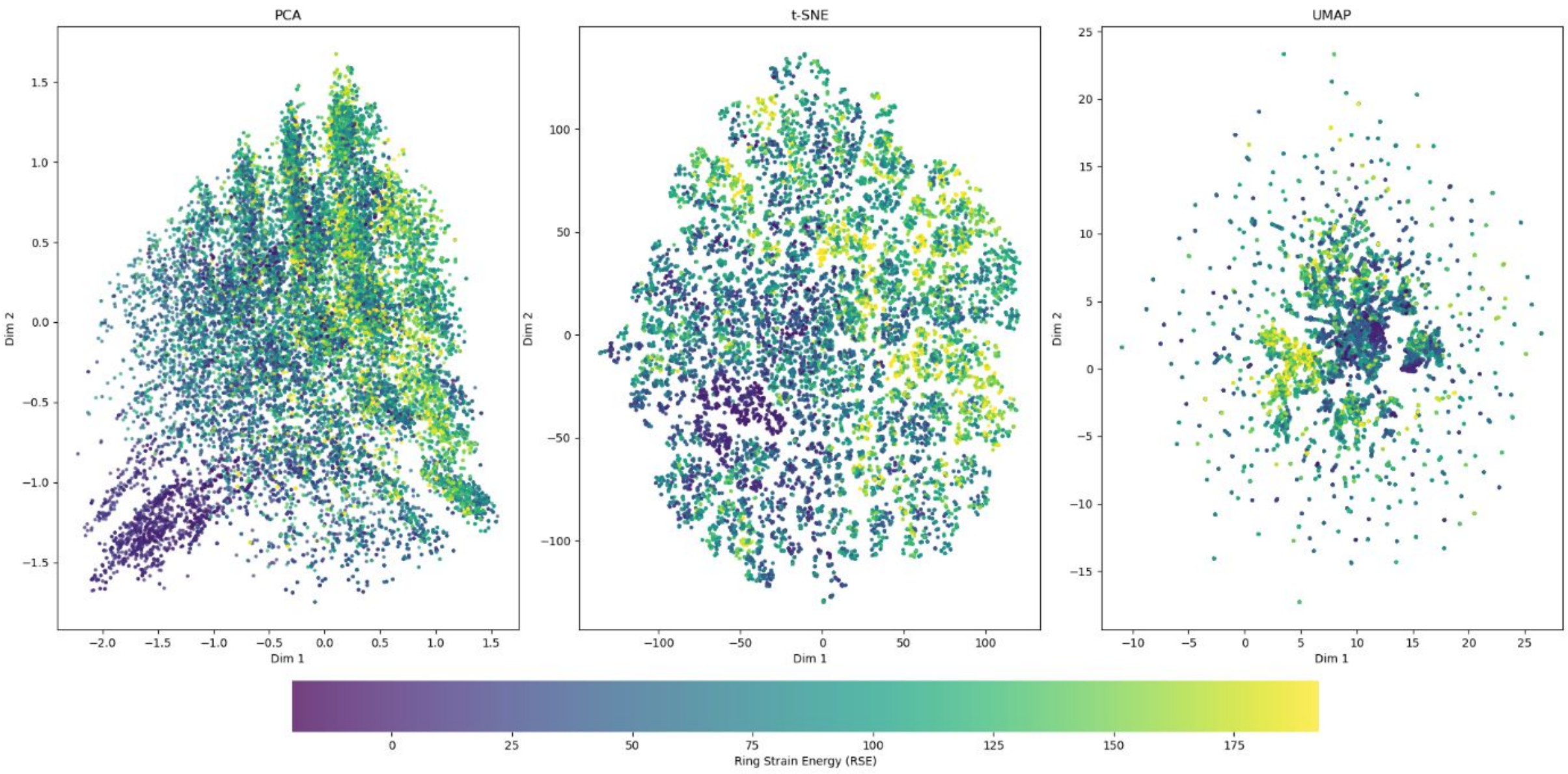

Figure S12: PCA, t-SNE and UMAP plots of the Morgan fingerprints for all molecules in CHX8. PCA was performed on the fingerprints with n_components = 2 and random_state = 42. For the t-SNE and UMAP plots a PCA was used to pre-process the data with n_components = 50 and random_state = 42 in both cases. t-SNE was generated with n_components = 2, perplexity = 40, init = 'pca', learning_rate = 'auto' and random_state = 42. UMAP was generated with n_neighbors = 50, min_dist = 0.1 and random_state = 42. A colour gradient is applied where each structure is coloured based on its calculated relative strain energy (RSE) / kcal mol^{-1}.

These plots not only give insight into the structural diversity contained within CHX8, but also give additional confidence in the success of the RSE metric. The PCA projection shows a clear RSE gradient from low to high strain across the two principle components, confirming that the structural features in this dataset are, as would be expected, intrinsically linked to their strain. The t-SNE and UMAP plots also demonstrate clean grouping of structures with different RSEs.

This provides clear evidence to the structural diversity of CHX8, suggesting that the dataset effectively maps a wide range of structural features, demonstrating a valuable platform for the investigation of novel, highly strained and unusual molecules.

References

1 B. D. McKay, *Congr. Numer.* 1981, ***30***, 45–87.
2 "RDKit: Open-source cheminformatics. https://www.rdkit.org, doi:10.5281/zenodo.825417," .
3 N. M. O'Boyle, M. Banck, C. A. James, C. Morley, T. Vandermeersch, G. R. Hutchison, *J. Cheminform.* 2011, **3**, 33.
4 S. R. Heller, A. McNaught, I. Pletnev, S. Stein, D. Tchekhovskoi, *J. Cheminform.* 2015, **7**, 23.
5 D. Weininger, "SMILES, a chemical language and information system. 1. Introduction to methodology and encoding rules" *J. Chem. Inf. Comput. Sci.* 1988, ***28***, 31–36.
6 A. Schaefer, H. Horn, R. Ahlrichs, *J. Chem. Phys.* 1992, ***97***, 2571–2577.
7 A. Schaefer, C. Huber, R. Ahlrichs, *J. Chem. Phys.* 1994, ***100***, 5829–5835.

8 F. Weigend, R. Ahlrichs, *Phys. Chem. Chem. Phys.* 2005, ***7***, 3297–3305.
9 F. Weigend, R. Ahlrichs, *Phys. Chem. Chem. Phys.* 2006, ***8***, 1057–1065.
10 M. Bursch, J. Mewes, A. Hansen, S. Grimme, *Angew. Chem. Int. Ed.* 2022, ***61***, e202205735.
11 F. Neese, *WIREs Comput. Mol. Sci.* 2012, ***2***, 73–78.
12 F. Neese, *WIREs Comput. Mol. Sci.* 2018, ***8***, e1327.
13 Jmol: an open-source Java viewer for chemical structures in 3D. http://www.jmol.org/.
14 Schrödinger Release 2024-1: Maestro, Schrödinger, LLC, New York, NY, 2024.
15 M. D. Hanwell, D. E. Curtis, D. C. Lonie, T. Vandermeersch, E. Zurek, G. R. Hutchison, *J. Cheminform.* 2012, ***4***, 17.
16 W. Maier, P. Schleyer, *J. Am. Chem. Soc.* 1981, **103**, 1981-1900.
17 J. Liebman, A. Greenberg, *Chem. Revs.* 1976, **76**, 311-365.
18 Wiberg, K. *Angew. Chem. Int. Ed.* 1986, **25**, 312-322.
19 B. M. Coia, L. A. Hudson, A. J. Specht, J. G. Kennemur, *J. Phys. Chem. A.* 2023, ***127***, 5005–5017.
20 In *'Strain' in IUPAC Compendium of Chemical Terminology, 5th Ed. Internation Union of Pure and Applied Chemistry; 2025. Online Version 5.0.0, 2025. https://doi.org/10.1351/goldbook.S06037*.
21 W. Hehre, R. Ditchfield, L. Radom, J. Pople, *J. Am. Chem. Soc.*1970, **92**, 4796-4801.
22 T. Dudev, C. Lim, *J. Am. Chem. Soc.* 1998, ***120***, 4450–4458.
23 R. D. Bach, O. Dmitrenko, *J. Am. Chem. Soc.* 2004, **126**, 4444-4452.
24 B. D. McKay, M. A. Yirik, C. Steinbeck, *J. Cheminform.* 2022, ***14***, 24.
25 D. Cremer, E. Kraka, H. Joo, J. A. Stearns, T. S. Zwier, *Phys. Chem. Chem. Phys.* 2006, ***8***, 5304.
26 C. A. Nixon, A. E. Thelen, M. A. Cordiner, Z. Kisiel, S. B. Charnley, E. M. Molter, J. Serigano, P. G. J. Irwin, N. A. Teanby, Y.-J. Kuan, *Astron. J.* 2020, ***160***, 205.
27 V. V. Gobre, S. P. Gejji, R. K. Pathak, *J. Phys. Chem. A.* 2022, ***126***, 5721–5728.
28 H. E. Matthews, W. M. Irvine, *Astrophys. J.* 1985, ***298***, L61–L65.
29 K. J. Daoust, S. M. Hernandez, K. M. Konrad, I. D. Mackie, J. Winstanley, R. P. Johnson, *J. Org. Chem.* 2006, ***71***, 5708–5714.
30 M. Ceylan, S. Yalçın, H. Seçen, Y. Sütbeyaz, M. Balci, *J. Chem. Res.* 2003, ***2003***, 21–23.
31 M. M. Yamano, A. V. Kelleghan, Q. Shao, M. Giroud, B. J. Simmons, B. Li, S. Chen, K. N. Houk, N. K. Garg, *Nature* 2020, ***586***, 242–247.
32 P. Saxe, H. F. Schaefer, *J. Am. Chem. Soc.* 1980, ***102***, 3239–3240.
33 R. A. Seburg, E. V. Patterson, J. F. Stanton, R. J. McMahon, *J. Am. Chem. Soc.* 1997, ***119,*** 5847–5856.
34 C. D. Sherrill, C. G. Brandow, W. D. Allen, H. F. Schaefer, *J. Am. Chem. Soc.* 1996, ***118***, 7158–7163.
35 K. Wiberg, S. Waddell. *J. Am. Chem. Soc.* 1990, 112, 2194-2216.
36 L. Blum, J-L. Reymond, *J. Am. Chem. Soc.* 2009, ***131***, 8732-8733.
37 L. McInnes, J. Healy, N. Saul, L. Großberger, *J. Open Source Softw.* 2018, **3**, 861.
38 K. Pearson, *Philos. Mag.* 1901*,* **2**, 559-572.
39 H. Hotelling, *J. Educ. Psychol.* 1933, **24**, 417-441.
40 L. v. d. Maaten, G. Hinton, *J. Mach. Learn. Res.* 2008, **9**, 2579-2605.
